# A review of the bioactive properties of Mongolian plants, with a focus on their potential as natural food preservatives

**DOI:** 10.1002/fsn3.3529

**Published:** 2023-07-05

**Authors:** Munkhjargal Burenjargal, Tuya Narangerel, Tuyagerel Batmunkh, Alideertu Dong, Saruul Idesh

**Affiliations:** ^1^ Department of Chemistry National University of Mongolia Ulaanbaatar Mongolia; ^2^ Department of Chemical and Biological Engineering National University of Mongolia Ulaanbaatar Mongolia; ^3^ College of Chemistry and Chemical Engineering, Engineering Research Center of Dairy Quality and Safety Control Technology, Ministry of Education Inner Mongolia University Hohhot China

**Keywords:** antibacterial, antifungal plants, antioxidant, Mongolian plants, natural food preservatives

## Abstract

Consumers have recently preferred food that is easy to make and of excellent quality, as well as food that is safe, natural, and minimally processed, but has a longer shelf life. Food deteriorates over time as a result of microbiological, chemical, or physical changes. Phytochemicals derived from medicinal and food plants have long been recognized for their biological activity to protect plants. These bioactivities are designed to increase the shelf life of food while inhibiting the growth of microorganisms. The use of natural plant food preservatives containing bioactive compounds as health‐promoting agents is particularly intriguing. Furthermore, due to their effectiveness against food spoilage and foodborne pathogens, natural plant‐origin antimicrobial compounds have been investigated as alternatives to synthetic antimicrobial compounds for preserving food quality. This review focused on the plant composition and properties that can be utilized as a natural food preservative, as well as the possibilities of using Mongolian medicinal plants.

## Graphical abstract

The use of natural plant food preservatives containing bioactive compounds as health‐promoting agents is particularly intriguing. This review focused on the plant composition used as a natural food preservative, as well as the possibility of using Mongolian medicinal plants.

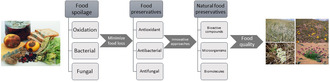

## 1 INTRODUCTION

Food losses are a major concern worldwide at the moment, as the world's population continues to grow, and nearly one‐third of all food produced for human consumption is either lost or wasted (FAO, 2011). As pathogenic microorganisms grow and lipid oxidation increases, the shelf life, safety, and quality of food are reduced (Campêlo et al., 2019). When considering food types, these losses correspond to 40%–50% of root crops, fruits, and vegetables; 35% of fish and seafood; 30% of cereals; and 20% of meat, oil seed, and dairy products (FAO, 2017).

The food industry is under significant strain as a result of the use of synthetic food additives such as artificial preservatives and antibacterial physical treatments that extend the shelf life of food by preventing bacterial growth. Today, consumers demand ready‐to‐eat, natural, microbiologically safe, and organic foods. Even short shelf life foods require special protection against bacterial contamination during their processing, storage, and transportation (Gutiérrez‐del‐Río et al., 2018).

Several food preservation methods are known, including traditional: drying, freezing, cooling, and pasteurization and advanced technologies, such as chemical processing, irradiation, high‐pressure technology, hurdle engineering, and nanotechnology. In addition, other procedures such as planting, harvesting, packaging, storing, and transporting are performed in a way to account for food preservation requirements (Amit et al., 2017; Bajpai et al., 2018; Msagati, 2013).

Living organisms, including plants, herbs, spice plants, animals, and microorganisms, produce a wide variety of secondary metabolites, such as terpenes, saponins, phenolic compounds, flavonoids, nitrogen‐ and sulfur‐containing compounds, thiosulfinates, and glucosinolates that exhibit antioxidative and antimicrobial activities. Because of these properties, they have been used as preservatives to replace artificial supplements and to increase the shelf life of a variety of foods or foodstuffs. In addition, these compounds have other valuable features, such as flavoring properties, etc., so they can be considered multifunctional (Amit et al., 2017; Campêlo et al., 2019; Msagati, 2013).

The present study reviews some Mongolian natural plants with antioxidant, antibacterial, and antifungal properties that can be used as food preservatives. There are 3160 flowering plant species registered, 1100 of which are medicinal plants used in Mongolian traditional medicine (Magsar et al., 2018). The herbal plants of Mongolia contain a variety of secondary metabolites, including tannins, dyes, coumarins, flavonoids, alkaloids, saponins, vitamins, essential oils, and alkaloids (Magsar et al., 2017).

Medicinal plants and spices have been used as therapeutics and food preservatives since antiquity worldwide, and there are some recognized ancient writings that record plant usage from a historical context. Traditional Asian medicines, particularly Chinese, Tibetan, and Ayurvedic medicine, are well known throughout the world and are used as alternative or complementary medicines (El‐Sayed & Youssef, 2019). The Mongolian Traditional Medicine originated from Mongolian, Tibetan, and Ayurvedic medicine. Following a period of stagnation, scientific research into traditional medicine has been gradually evolving (Pitschmann et al., 2013). The field had resumed by Soviet Russian scientists. Medicinal herbs were no exception.

The country is located at the crossroads of two major floristic regions of the world, the Siberian taiga and the Central Asian steppe and desert, and its topography encompasses a wide range of vegetation zones, including alpine, taiga, steppe, wetland, and desert. Its climate is also extreme, with temperatures ranging from +45°C to −45°C. Mongolian plants had to be adaptable in order to survive in these severe conditions, which required the production of various metabolites, which are the main ingredients in traditional medicines. In recent years, phytochemical studies on traditional medicinal plants used by Mongol nomadic have been conducted in Mongolia. However, phytochemical findings are preliminary and not comprehensive. The gathering and analysis of Mongolian folk medicinal botanical knowledge have grown in importance. As of 2013, 11 traditional herbal products had been officially approved, and many unregistered medicinal plants were widely used in our healthcare system (Pitschmann et al., 2013). For example, *Achillea asiatica* Serg., *Dianthus versicolor Fisch ex Link.*, *Euphorbia pallasii Turcz. ex Ledeb*, *Lilium pumilum Delile*, and *Saussurea amara* (L.) DC are all used in Traditional Mongolian Medicine to treat liver diseases. To date, medicinal and food plant research refers to the development of new products or alternative applications in the pharmaceutical and food industries (Gonchig et al., 2008).

## 2 ANTIOXIDANTS

The diversity of plant metabolites and their chemical structures, especially flavonoids, is associated with a wide variety of their biological activities. Their hydroxyl groups are efficient at scavenging free radicals, thus interrupting oxidative chain reaction. This makes these compounds powerful antioxidants (Gharras, 2009). Antioxidants support cellular defense mechanisms by reducing the risk of oxidative stress (Nimse & Pal, 2015). Aside from their enzymatic vs. nonenzymatic activity, antioxidants can be classified into several groups based on their activity, solubility in water/organic solvents, and size (low and high molecular; Nimse & Pal, 2015). There are five kinds of natural antioxidants: free radical terminators, metal chelators, singlet oxygen quenchers, oxygen scavengers, and antioxidant regulators (Carocho et al., 2015; Mathew & Abraham, 2006). The wide range of antioxidants is derived from a range of spice sources such as phenolic acids (gallic acid and caffeic acid), flavonoids (quercetin and kaempferol), phenolic diterpenes (carnosic acid and carnosol), and volatile essential oils (thymol and menthol; Shan et al., 2005). Some studies indicate that antioxidative activity of polyphenols is greater than that of synthetic antioxidants, such as BHA (butylated hydroxyanisole) and BHT (butylated hydroxytoluene). Even though natural antioxidants inhibit oxidation stress, their antioxidant potential depends on many other factors, such as food composition, matrix type (emulsion, foam, aqueous, and protein), the presence of other constituents, and environmental properties (ionic strength and pH; Brewer, 2011).

In Mongolia, *Carum carvi* L. is a wildly grown as a medicinal herb and vegetable. The previous studies revealed its antioxidant characteristics based on chemical compositions including monoterpene alcohols, linalool, carvacrol, carvone, limonene, anethole, estragole, flavonoids, and other polyphenolic compounds in caraway oil and aqueous extracts from roots (Agrahari & Singh, 2014). The major compounds in Mongolian caraway were found to be (S)‐(+)‐carvone (up to 65%) and (R)‐(+)‐limonene (up to 50%), with minor compounds also detected. It is an ingredient of the various traditional prescriptions and is used to treat nervous diseases, tumors, eye diseases, bronchial phlegm, inflammation, and stomach disorders. In addition, the antioxidant activity was found in some local medicinal plants, including *Chiazospermum erectum* Bennh (total alkaloid content, not less than 0.6%), which is used to treat typhoid fever, poisoning, and blood fever, as well as to relieve pain, reduce temperature, and expel bile. *Dianthus superbus* L. (total flavonoid content, not less than 1.2%), *Gentiana barbata* Froel (total alkaloid content 0.2%), *Hippophae rhamnoides* (Wild.) L., *Inula Britannica* L., *Odontites ruber* Gilib (total polyphenolic compound content, not less than 5.0%), *Oxytropis strobilacea* Bunge (rutin content, not less than 2.0%), *Polygonum hydropiper* Lour, *Vaccinium vitis‐idaea* L. (tannins, not less than 5.0%), and *Zygophyllum potanini* Maxim (total flavonoid content based on kaempferol, 1.4%; WHO, 2013).

*Allium mongolicum* Regel is also well distributed in the country as a medicinal herb, which has some biological activities such as antioxidant, antitumor, antihypertensive, antimicrobial, anti‐inflammatory, and improver gastrointestinal motility, hypolipidemic, appetite stimulating, kidney replenishing, and aphrodisiac (Dong et al., 2020; Wang et al., 2019). Wang et al. (2019) have studied the bioactivities of *A. mongolicum* in aqueous and methanol extracts. As reported by Wang et al. (2019), *A. mongolicum* has shown strong antioxidant activity.

Moreover, the strong antioxidant activities of *Fragaria orientalis* Losinsk, *Vaccinium vitis‐idaea*, *Hippophae rhamnoides*, and *Paeonia anomala* L. were discovered that ranged from 797, 923, 1300 to 1544 mol TE/g dry leaf, respectively. The highest total phenolic compounds and flavonoid contents were observed in the dry leaf of the *Vaccinium vitis‐idaea* (175 and 9 mg QE/g) (Enkhtuya et al., 2014). Birasuren et al. (2012) documented the antioxidant activity of *Ribes diacanthum* Pall. in its various extracts, which have traditionally been used in Mongolian medicine to treat kidney illnesses such as cystitis, bladder infections, retention, and edema. The ethyl acetate extract of *R. diacanthum* Pall. has potential antioxidant activities (Birasuren et al., 2012).

The lowest value with a radical scavenging capacity of 50% expresses the powerful antioxidant characteristics of the medicinal plants. Based on this value, *Rhodiola rosea* L., *Saxifraga Spinulosa* Adams, *Bupleurum* L., *Artemisia dolosa* Krasch. and *Cicuta virosa* L. were indicated to have strong antioxidant properties (5.84, 28.7, 67.93, 103.96, and 165.14 μg/mL, respectively; Badral et al., 2017).

## 3 ANTIBACTERIALS

The second group of natural food preservatives is antibacterial plants, which contain terpenes, peptides, polysaccharides, and phenolic compounds. These bioactive compounds have medicinal characteristics including antioxidant, antimicrobial, and anti‐inflammatory effects that reduce or inhibit the risk of chronic and acute diseases. The primary function of antimicrobial activity is to control the growth of microorganisms that cause bacterial and fungal contamination in food products. The main goal of their use in food industry is to prevent food spoilage in processed and ready‐to‐eat food.

Microorganisms can have an impact on food in both positive and negative ways. *Saccharomyces cerevisiae*, lactic acid bacteria, and *Acetobacterium* species are the main microorganisms for food fermentation (Xu, 2017). Several phenolic compounds as well as ferulic acid, coumaric acid, and catechins were found to inhibit the growth of microbes, for example, *Staphylococcus aureus*, *Salmonella enteritidis*, and *Listeria monocytogenes* (El‐Sayed & Youssef, 2019). Saprophytic microorganisms also influence the spoilage of food and the growth of pathogenic microorganisms that cause food‐borne illnesses (Xu, 2017). Many natural ingredients have antimicrobial properties that range from mild to potent. An ideal model compound for the natural antimicrobial activity should have broad bactericidal and fungicidal activities, be active at low concentrations, heat stable, unaffected by pH, nontoxic, easily assayable, nonpharmaceutical, nonsusceptible to resistance from contaminants, label‐friendly, and cost‐effective (Carocho et al., 2015).

The primary function of secondary metabolites with antimicrobial activity is to protect against pests and stress while also acting as signaling molecules between the cell and the environment. The mixture of plant polyphenols and essential oils with antimicrobial properties is used for the preservation of raw and processed products, for example, meat, fish, dairy products, fruit, and vegetables. Furthermore, essential oils such as thymol and carvacrol have been used in the packaging of food (Carocho et al., 2015).

The research on the antibacterial activity of Mongolian herbs is summarized in Table 1. Antibacterial activity of bacteria such as *S. aureus*, *E. faecalis*, and *M. luteus* was determined in most plants.

**TABLE 1:** The antibacterial activity of some Mongolian plants (inhibited growth in mm).

Plant names	*S. aureus*	*E. coli*	*P. aeruginosa*	*N.Gonorrhoeae*	*C.albicans*	*S. epidermidis*	*E. faecalis*	*M. luteus*	*B. subtilis*	*B. cereus*	*C. violaceum*	References
*Abies sibirica L.*	13.1							22				Gonchig et al. (2008)
*Agrimonia pilosa L.*	20.2							21.3				
*Aquilegia sibirica Lam.*	11.2											
*Artemisia pectinata Pall.*	14.7							18				
*Astragalus junafovii* Sancz.											19–25	(Zolboo et al., 2015)
*Caryopteris mongolica* Bunge.	10.2–12.9					10.2–15.9	9.6–23.2	9.0–16.3				(Saruul et al., 2015)
*Comarum salesovianum Steph.*	24.2						15.3	24.7				Gonchig et al. (2008)
*Dasiphora fruticosa (L.) Rydb.*	11.5–13.0						10.2	12.6–13.0				
*Goniolimon speciosum (L.) Boiss.*	18							10.9			23.5–27.5	Gonchig et al. (2008)
*Hedysarum alpinum* L.											24.5	Zolboo et al. (2015)
*Lagotis integrifolia (Willd.) Schischk.*	11.9							11.4				Gonchig et al. (2008)
*Ledum palustre* L.											19–28	Zolboo et al. (2015)
*Lophanthus chinensis (Raf.)* Benth.											22	
*Lophanthus krylovii* Lipsky.											20.5	
*Malva mohileviensis Down.*								11.6				Gonchig et al. (2008)
*Myricaria alopecuroides Schrenk.*	11.7						12.2	13.7				
*Paeonia anomala L.*	16.5	10					12	25.7				
*Pinus sylvestris. var mongolica* Litv.	4								5	5		Namshir et al. (2020)
*Potentilla viscosa G. Donn*	14						14.3	15				Gonchig et al. (2008)
*Pyrola incarnata (D.C.) Freyn*	17.5						13.6	18.4				
*Pyrethrum pulchrum* L	11		17		12		14		11			Erdenetsogt et al. (2019)
*Rhinanthus songaricus (Skerneck) B.Fedtsch.*								9.8				Gonchig et al. (2008)
*Rosa acicularis Lindl.*	13.2						12.4	15.8				
*Scutellaria baicalensis Georgi*	15.9											Gonchig et al. (2008)
*Sedum aizoon L.*	13.6						9.7	11.9				Gendaram et al. (2011); Gonchig et al. (2008)
*Sedum hybridum L.*	10.5; 8.6–13.9	9.2–13.5					17.1; 9.2–13.2	14.6; 9.1–15.8	15.8			Gonchig et al. (2008)
*Serratula centauroides L.*								10				
*Spiraea media* F. Schmidt											18	Zolboo et al. (2015)
*Spongiocarpella grubovii* Ulzii											36.5	

From the table, *Sedum hybridum* L. (Crassulaceae), a local medicinal plant, demonstrated strong antioxidant (31.93 ± 0.65 μg/mL at IC_50_) and antibacterial activities in ethanol extract against gram‐positive strains *M. luteus* and *S. aureus*. Herbs for food preservation can be utilized as whole plants, in parts, and in various forms such as extracts, solutions, and dried forms. As a result, it is appropriate to utilize the plant parts containing the most biologically active chemicals. Eighteen known compounds were extracted and identified using serial column chromatography of the ethyl acetate fraction of the plant extract using several packing materials and the researchers also investigated the antibacterial properties of isolated compounds found in medicinal plants against bacterial strains *P. aeruginosa*, *E. coli*, *E. faecalis*, *M. luteus*, and *S. aureus* by the disk diffusion method. The ethyl gallate inhibited the growth of *M. luteus*. (10.3–15.8 mm), *E. faecalis* (9.2–13.2 mm), *E. coli*, (9.2–13.5 mm), and *S. aureus* (9.8 mm) at 200–1000 μg/disk using the disk diffusion method (Gendaram et al., 2011). In 2015, Saruul et al. (2015) conducted the similar study. Aromatic abietane diterpenoids (demethylcryptojaponol, 6α‐hydroxydemethyl cryptojaponol, and 6‐hydroxysalvinolone) isolated from *Caryopteris mongolica* suppressed the growth of the *S. aureus*, *S. epidermidis*, *E. faecalis*, *and M. luteus*.

Moreover, natural antimicrobial – essential oil of the *Pyrethrum pulchrum* Ledeb. was investigated by Erdenetsogt et al. (2019). The essential oil, which was mostly composed of camphor (33.9%), linalool (21.1%), and α‐pinene (9.0%), indicated a medium antibacterial activity against bacteria *P. aeruginosa*, *M. vaccae*, *and E. faecalis*. As previously reported, the essential oil extracted from the aerial part of the plant produced the same results (Erdenetsogt et al., 2019).

In 2008, Gonchig et al. conducted a large study on the antibacterial effects of the plants' extracts collected from 67 different plant species. The whole herb extracts of *Hedysarum alpinum*, *Comarum salesovianum*, and *Sedum hybridum* exhibited significant antibacterial activity against *P. aeruginosa* (10.5 mm), *S. aureus* (24.2 mm), and *E. faecalis* (17.1 mm), respectively. Furthermore, the root extract of *Paeonia anomala* exposed the highest antibacterial properties against *E. coli* (10 mm) and *M. luteus* (25.7 mm). Besides, the antibacterial activities of the plant extracts were evaluated in the different solvent fractions (dichloromethane, ethyl acetate, n‐butanol, n‐hexane, and water) and concentrations (0.5–4 mg/disc). The strong antibacterial activities were observed for ethyl acetate and n‐butanol fractions obtained from the whole herb of *Sedum hybridum* and *Sedum aizoon* as well as the ethyl acetate fraction of the leaf extract of *Dasiphora fruticosa* and root extract of *Paeonia anomala* against *S. aureus* and *M. luteus*, respectively. Moreover, the ethyl acetate and n‐butanol fractions of flower extracts of the *Dasiphora fruticosa* and *Sedum aizoon* were found to have a clear antibacterial effect against *E. faecalis*, while the whole herb extract of *Sedum aizoon* at 2 and 4 mg/disc demonstrated effective antibacterial activity against *S. aureus* (Gonchig et al., 2008). Antibacterial properties were also found in *Chelidonium majus* L*. and Plantago major* L. (WHO, 2013). Plants with the highest antibacterial activity, such as *Sedum hybridum*, *Pyrethrum pulchrum L*, and *Caryopteris mongolica Bunge*, have the potential to be used as natural food preservatives in the future.

## 4 ANTIFUNGALS

Fungi, among spoilage microorganisms, are a major issue at any stage of the food chain because of their ability to grow in a variety of, even harsh environments. As a result, food spoiling by mold can result in significant economic losses for the food industry, as well as a health risk for consumers due to mycotoxin‐producing mold species (Pitt & Hocking, 2013).

Beyond their negative impact on food quality, some fungal genera such as *Aspergillus*, *Penicillium*, *Alternaria*, and *Fusarium* have the ability to produce mycotoxins that can have a toxic effect on humans and animals. Furthermore, mycotoxins are able to withstand many food processing steps, raising concerns about food safety (Sanzani et al., 2016). *A. ochraceus* and *P. verrucosum*, *A. westerdijkiae* are considered the most important ochratoxin A (OTA) producers in foods (Frisvad et al., 2019; Mantle, 2002). OTA is nephrotoxic, neurotoxic, hepatoxic, teratogenic, genotoxic, immunotoxic, embryotoxic, and carcinogenic and is classified as a possible human carcinogen (group 2B; Mantle et al., 2015; WHO, 2008). OTA is not destroyed during standard food production processes due to its high stability and can be found both in raw materials and in processed foods such as cereals and cereal products, coffee, grapes, wine, beer, cocoa, nuts, dried fruits, and spices (Jeršek et al., 2014; Malir et al., 2016).

A conventional way to prevent decomposition by microorganism growth is the use of preservatives. Despite the desire for safe food, there is an increasing consumer demand to avoid or diminish chemical food additives, resulting in a growing interest in new safe and biodegradable preservatives. The possible adverse health and ecological effects of convinced fungicides and preservatives have led to more natural methods. Until now, many plant extracts with antibacterial and antifungal activity have already been known, including essential oils (EOs) and their active components (Bakkali et al., 2008; Calo et al., 2015).

Giordani et al. (2020) investigated the antifungal activity of extracts obtained from some Mongolian medicinal plants by measuring their minimal inhibitory concentration (MIC) against fungi cause of cutaneous diseases such as *Candida* species, dermatophytes *Microsporum gypseum*, *Trichophyton mentagrophytes*, and *Malassezia furfur*. Only *Stellaria dichotoma* L., *Scutellaria scordifolia* L., *Aquilegia sibirica* Fisch. Et Schrenk, and *Hyoscyamus niger* L. showed antifungal activity. Their geometric mean (GM) MIC_50_ values against *Candida* spp were 84, 22, 181, and 169 mg/mL, respectively. The GM MIC_50_ values for dermatophytes of *Scutellaria scordifolia* L., *Aquilegia sibirica* Fisch. Et Schrenk were 32 and 256 mg/mL, correspondingly. For *Malassezia furfur*, *Scutellaria scordifolia* L. showed the best activity with MIC_50_ value of 64 mg/mL. In particular, *S. scordifolia* L. methanol extract exhibited the best activity against *Candida* spp., dermatophytes, and *Malassezia furfur* with GM MIC_50_ values of 22, 32, and 64 mg/mL, respectively (Olennikov & Chirikova, 2013; Shang et al., 2010).

Several studies have shown that *Scutellaria barbata*'s essential oil inhibits the growth of *Candida albicans*, *Candida tropicalis*, and *S. aureus* (Yu et al., 2004) and *Scutellaria baicalensis* L. plant extracts prevent the growth of *C. albicans*, *Cryptococcus neoformans*, *Pityrosporum ovale*, and *S. aureus* (Gonchig et al., 2008). *Scutellaria barbata* has 51 flavonoids (Каримов et al., 2016. *Scutellaria barbata* is most noted for its potent anticancer properties and is frequently combined with other herbs for anticancer traditional Chinese medicine formulations to treat cancers of the lung, liver, breast, and gastrointestinal tract (Dharmananda, 2004; Gao et al., 2019). To date, multiple classes of phytochemicals, including flavonoids, essential oils, polysaccharides, and terpenoid alkaloids, have been identified from *S. barbata* herb (Gao et al., 2019). The most widespread and therefore studied in detail is *S. baicalensis*. So far, 131 flavonoids, 96 flavone derivatives, 21 flavanones, 6 flavonols, 4 isoflavones, 3 flavanonols and 1 chalcone have been found in various organs of *S. baicalensis* (Каримов et al., 2016)

Currently, 57 natural compounds isolated from 16 plant species of *Aquilegia* genus. These include labdane diterpene −2 (3.5%), cycloartane −13 (22.8%), flavonoids −22 (38.5%), alkaloids and nitrogen‐containing compounds −10 (17.5%) and phenolic acids, and fatty acids. From these 19 new natural compounds belonging to cycloartane glycosides, labdane diterpene, flavoalkaloids, and nitrile nitrogen compound have been identified. The presence of flavoalkaloid can consider a specific characterization of *Aquilegia* species (Odontuya & Nomin, 2018).

The flavones luteolin and apigenin identified in *S. scordifolia* Fisch. ex Schrank extracts, as well as rutin found in *Stellaria dichotoma* L. and *Hyoscyamus niger* L. extracts, could be responsible for the observed antifungal activity (Giordani et al., 2020). Dulger et al. (2010) studied the antifungal properties of the methanolic extract obtained from *H. niger* against some clinically relevant fungi such as *Candida* and *Cryptococcus* species. Greater activity was observed for the extracts of *H. niger* against both *Cryptococcus* species, with values of 15 μg/mL. The extracts have a strong effect against *Candida albicans* and *C. guilliermondii* as the same MIC values (60 μg/mL), followed by yeast cultures such as *C. tropicalis*, *C. krusei*, and *C. parapsilosis* have susceptible to the extract at a MIC of 12.5 μg/mL. The highest MICs of the extract were 250 μg/mL against *C. glabrata* (Dulger et al., 2010).

Table 2 lists the Mongolian herbs that have been examined and determined for their biological activity useful in food preservation, as well as their chemical components.

**TABLE 2:** Mongolian herbs with antioxidant, antimicrobial, and antifungal properties.

Herbs	Compounds	Antioxidant	Antibacterial	Antifungal	Authors
*Calligonum mongolicum Turcz.*	22 compounds	+	+		Buyankhishig et al. (2021)
*Caryopteris mongolica Bunge*	7 compounds		+		Saruul et al. (2015)
Leaves of *Paeonia anomala L.*	Methyl gallate, pentagalloylglucose, tellimoside	+			Enkhtuya et al. (2017)
Mongolian medicinal plant extracts	‐		+	+	Enkhtuya et al. (2017)
Mongolian medicinal plants	‐		+		Gonchig et al. (2008)
*Oxytropis pseudoglandulosa Gontsch. ex Grubov*	26 components in volatile oil and 21 compounds in lipid extract	+	+		Narangerel, Bonikowski, et al. (2021)
*Pinus sylvestris. var mongolica* Litv.	101 compounds in the essential oil.	+	+		Namshir et al. (2020)
*Pyrethrum pulchrum* Ledeb.	55 chemical compounds in essential oil.		+	+	Erdenetsogt et al. (2019)
*Ribes diacanthum* Pall	‐	+			Birasuren et al. (2012)
*Saxifraga spinulosa Adams*	25 compounds in aerial parts.	+	+		Birasuren et al. (2012)
*Sedum hybridum* L. (*Crassulaceae*),	18 compounds	+	+		Gendaram et al. (2011)
*Thymus baicalensis Serg.*	37 chemical compounds in volatile oil, 10 polyphenolic compounds, and 32 lipid components		+		Narangerel, Sójka, et al. (2021)

In addition, Table 3 contains the most prevalent local herbal spices that can be used as natural food preservatives. The antioxidant, antibacterial, and antifungal activities of commonly used herbal spices in Mongolia are listed in Table 3 on the basis of some applications including raw and processed food preservation, alternative medicines, and natural therapies. The relationship between antioxidant properties of spices and food spoilage has been well documented. Starting from the food preparation, spices can affect both food spoilage microorganisms (food preservation) and human pathogens (food safety) due to the antibacterial and antifungal activity of their natural constituents. These species can be used as sources of natural food preservatives with remarkable health benefits. Several studies have documented these effects and, in some cases, confirmed the mechanisms of action, though more research is needed in this area (Carocho et al., 2014).

**TABLE 3:** Herbal spices with antioxidant, antibacterial, and antifungal activities as natural food preservatives.

Mongolian herbal spices	Bioactive properties			Main biological compounds
	Antioxidant	Antibacterial	Antifungal	
*Allium sativum* L.‐Garlic	+	+	+	Organosulfur such as allicin, phenolic, and polysaccharides compounds (Avato et al., 2000; Bag & Chattopadhyay, 2015; Batiha et al., 2020; Lanzotti, Barile, et al., 2012; Leontiev et al., 2018; Martins et al., 2016; Medina et al., 2019; Pârvu et al., 2019; Prados et al., 2005; Shams‐Ghahfarokhi et al., 2006; Szychowski et al., 2018)
*Allium cepa* L.‐Onion	+	+	+	Thiosulfinate compounds, phenolic compounds, especially flavonoids, and several organosulfur compounds, polysaccharides, quercetin, quercetin‐4′‐monoglucoside, quercetin‐3,4′‐diglucoside, anthocyanins, and essential oils (Bag & Chattopadhyay, 2015; Fredotovíc et al., 2017; Kocić‐Tanackov et al., 2017; Lanzotti, Romano, et al., 2012; Ma et al., 2018; Marefati et al., 2021; Prakash et al., 2007; Sharma et al., 2014)
*Allium mongolicum* Regel‐Mongolian onion	+	+	+	Polysaccharides, flavonoids polyphenols, and phenols (Chen et al., 2020; Ding et al., 2021; Li, Guo, et al., 2019; Li, Zhu, et al., 2019; Wang et al., 2019)
*Allium senescens* L.‐ Aging chive	+	+	+	Organosulfur compounds, saponins, and phenolics (Kyung, 2012; Pârvu et al., 2011; Qin et al., 2021
*Allium victorialis* L.‐ Alpine leek	+	+	+	Allicin, kaempferol, ferulic acid, astragalin, methyl allyl disulfide, quercetin, diallyl disulfide, and furostanol glycosides (Khan et al., 2015; Kyung, 2012; Nishimura et al., 2006; Woo et al., 2012
*Artemisia dracunculus L.‐*Tarragon	+	+	+	L‐ascorbic acids, chlorophylls, carotenoids, tannins and flavonoids, essential oils, coumarins (>1%), flavonoids, and phenol carbonic acid (Kordali et al., 2005; Nurzynska‐Wierdak & Zawislak, 2014; Obolskiy et al., 2011; Parham et al., 2020; Sandulachi et al., 2021; Souri et al., 2004; Zare et al., 2021)
*Brassica juncea* L Czern*.‐*Mustard	+	+	+	Phenolics, sinapic acid and sinapoyl esters, glucosinolate (Engels et al., 2012; Kurt et al., 2011; Miceli et al., 2014; Oguro et al., 2014; Park et al., 2017; Sturm & Wagner, 2017; Sun et al., 2018; Thiyam, Stöckmann, & Schwarz, 2006; Thiyam, Stöckmann, Zum Felde, et al., 2006; Tian & Deng, 2020
*Carum carvi* L*.‐*Caraway	+	+	+	Essential oils—carvone, limonene, germacrene D, and trans‐dihydrocarvone, (Assami et al., 2012; Hajlaoui et al., 2021; Hromiš et al., 2015; Kocić‐Tanackov et al., 2020; Obolskiy et al., 2011; Rasooli & Allameh, 2016; Samojlik et al., 2010; Seidler‐Łozykowska et al., 2013; Tomović et al., 2021)
*Coriandrum sativum* L*.‐*Coriander	+	+	+	Essential oils—(+)‐Linalool (S)‐(+)‐linalool, α‐Pinene β‐Pinene, γ‐Terpinene, α‐Cedrene (Alves‐Silva et al., 2013; Bogavac et al., 2015; Delaquis et al., 2002; Diana et al., 2016; Dua et al., 2014; Freires et al., 2014; Kačániová et al., 2020; Laribi et al., 2015; Mandal & Mandal, 2015; Marangoni & De Moura, 2011; Sriti et al., 2011)
*Foeniculum vulgare Mill.‐*Fennel	+	+	+	Phenolic compounds, essential oils such as (E)‐anethole, methyl‐chavicol, fenchone, and limonene, phenylpropanoid derivatives, monoterpenoids, and sesquiterpene hydrocarbons (Anwar et al., 2009; Badgujar et al., 2014; Belabdelli et al., 2020; Ben Abdesslem et al., 2021; Choi & Hwang, 2004; Conforti et al., 2006; Diao et al., 2014; Farid et al., 2020; Goswami & Chatterjee, 2014; He & Huang, 2011; Kaur & Arora, 2009; Mohamad et al., 2011; Morales et al., 2012; Roby et al., 2013; Sayed‐Ahmad et al., 2017; Shahat et al., 2011)
*Glycyrrhiza glabra L.‐*Licorice	+	+	+	Triterpenes, saponins (responsible for the sweet taste), flavonoids, glycyrrhizin, 18β‐glycyrrhetinic acid, glabridin A and B, and isoflavones, dihydrostilbene derivatives, and isoflavans (Ajagannanavar et al., 2014; Alagawany et al., 2019; Ali, 2013; Cheema et al., 2014; Chouitah et al., 2011; Gupta et al., 2008; Hosseinzadeh & Nassiri‐Asl, 2015; Irani et al., 2010; Jang et al., 2008; Karahan et al., 2016; Karkanis et al., 2018; Kim et al., 2013; Messier & Grenier, 2011; Pastorino et al., 2018; Simmler et al., 2013; Singh et al., 2015; Siracusa et al., 2011)
*Malva sylvestris*. L ‐Mallows	+	+		Polyphenols, anthocyanins, coumarins, tannins, flavonoids, flavones, flavonols, anthocyanidines, leucoanthocyanidines, mucilage, and terpenoids such as sesquiterpenes, diterpenes, monoterpenes (Barros et al., 2010; Cecotti et al., 2016; Cheng & Wang, 2006; Feizi et al., 2018; Mohammed et al., 2014; Mousavi et al., 2021; Razavi et al., 2011; Sharifi‐Rad et al., 2020; Zhen‐yu, 2005)
*Matricaria recutita* L.‐ Chamomile	+	+	+	Flavonoids, terpenoids, and phenolic compounds primarily the flavonoids apigenin, quercetin, patuletin, luteolin and their glucosides, apigenin, and matricin (Al‐Dabbagh et al., 2019; Höferl et al., 2020; Jang et al., 2008; Kazemi, 2015; Miraj & Alesaeidi, 2016; Sánchez et al., 2020; Sharifi‐Rad et al., 2018; Singh et al., 2011; Stanojevic et al., 2016; Tsivelika et al., 2021)
*Mentha arvensis* L.‐Pennyroyal	+	+		Menthol, somenthone/neoisomenthol, neomenthol, menthone, phenolic acids (e.g., rosmarinic and caffeic acids), flavones (e.g., luteolin derivatives), and flavanones (e.g., eriocitrin derivatives; Anwar et al., 2019; Benabdallah et al., 2016; Biswas et al., 2014; Bokhari et al., 2016; Dorman et al., 2003; Heydari et al., 2018; Hussain et al., 2010; Kalemba & Synowiec, 2020; Khan et al., 2019; Kurilov et al., 2009; Pandey et al., 2003; Salehi et al., 2018; Singh & Pandey, 2018; Varma & Dubey, 2001)
*Mentha* L.‐Mint	+	+	+	Phenolic compounds, flavonoids (specific lipophilic flavonoids), terpenes/essential oils [(Iseppi et al., 2020; Jianu et al., 2021; Ludwiczuk et al., 2016; Mimica‐Dukic et al., 2003; Mimica‐Dukic & Bozin, 2008; Tafrihi et al., 2021; Zeljković et al., 2021)]
*Origanum vulgare* L.‐Oregano	+	+	+	Essential oil (with carvacrol and/or thymol, linalool, γ‐terpinene, and p‐cymene), polyphenols (flavonoids and phenolic acids), triterpenoids, and sterols (Bhargava et al., 2015; Chishti et al., 2013; De Falco et al., 2013; El Babili et al., 2011; Fliou et al., 2020; Gutiérrez‐del‐Río et al., 2018; Hernández‐Hernández et al., 2014; Koldaş et al., 2015; Lombrea et al., 2020; Lukas et al., 2015; Moghrovyan et al., 2019; Oniga et al., 2018; Parra et al., 2021; Pezzani et al., 2017; Pitaro et al., 2013; Potente et al., 2020; Prakash et al., 2021; Scalas et al., 2018; Soltani et al., 2021; Teixeira et al., 2013; Węglarz et al., 2020; Zhang et al., 2014)
*Portulaca oleracea* L.‐Portulaca	+	+		Ascorbic acid, a‐tocopherols, omega‐3 fatty acids, apigenin, gallotannins, quercetin, and kaempferol, alkaloids, terpenoids, flavonoids, and organic acids (Alam et al., 2014; Banerjee & Mukherjee, 2002; Fernández‐Poyatos et al., 2021; Gatea et al., 2017; Iranshahy et al., 2017; Rahimi et al., 2019; Sicari et al., 2018; Siriamornpun & Suttajit, 2010; Zhou et al., 2015)
*Thymus* L*.‐*Thyme	+	+	+	Phenolic acids and flavonoids, caffeic, rosmarinic, p‐coumaric acids, luteolin 7‐O‐glucoside, naringenin, and (−)‐epicatechin, carvacrol, thymol, γ‐terpinene, p‐cymene, linalool, and phenols (Ateeq‐Ur‐Rehman et al., 2009; Bogavac et al., 2015; Dauqan & Abdullah, 2017; De Martino et al., 2009; Fliou et al., 2020; Galovičová et al., 2021; Iseppi et al., 2019; Mandal & DebMandal, 2016; Marques et al., 2015; Mathlouthi, 2003; Neidhart, 2016; Nzeako et al., 2006; Paaver et al., 2008; Purcell et al., 2016; Scalas et al., 2018; Sefidkon et al., 2004)
*Tribulus terrestris* L.‐Tribulus	+	+		Tannin and phenolic acids, flavonoids, flavonol glycosides, steroidal saponins, and alkaloids (Al‐Bayati & Al‐Mola, 2008; Chhatre et al., 2014; Parham et al., 2020; Sivapalan, 2016; Wang et al., 2016; Zeeshan Amanullah et al., 2021)
*Urtica dioica L.‐*Nettle	+	+	+	Carotenoids, flavonoids, quercetin, and polyphenolics (Dar et al., 2013; Grauso et al., 2020; Majedi et al., 2021; Modarresi‐Chahardehi et al., 2012; Parham et al., 2020; Salehzadeh et al., 2014)

## 5 CONCLUSION

Plant phytochemicals have long been known to protect plants from bacteria, fungi, viruses, and herbivores, but it was lately revealed that they are also important in protecting humans from disease. It is well known that a diverse array of herbs, vegetables, fruits, and grains, besides having nutrients, vitamins, and minerals, also possess a large variety of biologically active compounds. Significant part of medicinal plants is consumed by humans, and as a food, it additionally improves human health and well‐being in general. The application of natural plant food preservatives with additional potential as health‐promoting agents is especially interesting.

Furthermore, because a number of the studies use data from species collected in the wild under uncontrolled conditions, more research is needed to understand the effects of preserving food of bioactive compounds and to enhance the use of this valuable genetic material.

## AUTHOR CONTRIBUTIONS

**Munkhjargal Burenjargal:** Data curation (equal); investigation (equal); resources (equal); software (equal); writing – original draft (equal). **Saruul Idesh:** Formal analysis (equal); methodology (equal); project administration (equal); supervision (equal); writing – original draft (equal); writing – review and editing (equal). **Tuya Narangerel:** Data curation (equal); investigation (equal); methodology (equal); validation (equal); writing – original draft (equal). **Tuyagerel Batmunkh:** Funding acquisition (equal); validation (equal); visualization (equal). **Alideertu Dong:** Methodology (equal).

## CONFLICT OF INTEREST STATEMENT

The authors declare that they do not have any conflict of interest.

## Data Availability

The data that support the findings of this study are available on request from the corresponding author.

## References

[fsn33529-bib-0001] Agrahari, P. , & Singh, D. K. (2014). A review on the pharmacological aspects of *Carum carvi* . Journal of Biology and Earth Sciences, 4(1), M1–M13.

[fsn33529-bib-0002] Ajagannanavar, S. L. , Battur, H. , Shamarao, S. , Sivakumar, V. , Patil, P. U. , & Shanavas, P. (2014). Effect of aqueous and alcoholic licorice (*Glycyrrhiza glabra*) root extract against *Streptococcus mutans* and *lactobacillus acidophilus* in comparison to chlorhexidine: An *in vitro* study. Journal of International Oral Health, 6(4), 29–34. 25214729 PMC4148569

[fsn33529-bib-0003] Alagawany, M. , Elnesr, S. S. , Farag, M. R. , El‐Hack, M. E. A. , Khafaga, A. F. , Taha, A. E. , Tiwari, R. , Yatoo, M. I. , Bhatt, P. , Marappan, G. , & Dhama, K. (2019). Use of licorice (*Glycyrrhiza glabra*) herb as a feed additive in poultry: Current knowledge and prospects. Animals, 9(8), 536. 10.3390/ani9080536 31394812 PMC6720273

[fsn33529-bib-0004] Alam, M. A. , Juraimi, A. S. , Rafii, M. Y. , Abdul Hamid, A. , Aslani, F. , Hasan, M. M. , Mohd Zainudin, M. A. , & Uddin, M. K. (2014). Evaluation of antioxidant compounds, antioxidant activities, and mineral composition of 13 collected purslane (*Portulaca oleracea* L.) accessions. BioMed Research International, 2014, 6–10. 10.1155/2014/296063 PMC3918865 24579078

[fsn33529-bib-0005] Al‐Bayati, F. A. , & Al‐Mola, H. F. (2008). Antibacterial and antifungal activities of different parts of *Tribulus terrestris* L. growing in Iraq. Journal of Zhejiang University: Science B, 9(2), 154–159. 10.1631/jzus.B0720251 18257138 PMC2225498

[fsn33529-bib-0006] Al‐Dabbagh, B. , Elhaty, I. A. , Elhaw, M. , Murali, C. , Al Mansoori, A. , Awad, B. , & Amin, A. (2019). Antioxidant and anticancer activities of chamomile (*Matricaria recutita* L.). BMC Research Notes, 12(1), 1–8. 10.1186/s13104-018-3960-y 30602390 PMC6317209

[fsn33529-bib-0007] Ali, E. M. (2013). Phytochemical composition, antifungal, antiaflatoxigenic, antioxidant, and anticancer activities of *Glycyrrhiza glabra* L. and *Matricaria chamomilla* L. essential oils. Journal of Medicinal Plants Research, 7(29), 2197–2207. 10.5897/JMPR2013.5134

[fsn33529-bib-0008] Alves‐Silva, J. M. , Dias dos Santos, S. M. , Pintado, M. E. , Pérez‐álvarez, J. A. , Fernández‐López, J. , & Viuda‐Martos, M. (2013). Chemical composition and *in vitro* antimicrobial, antifungal and antioxidant properties of essential oils obtained from some herbs widely used in Portugal. Food Control, 32(2), 371–378. 10.1016/j.foodcont.2012.12.022

[fsn33529-bib-0009] Amit, S. K. , Uddin, M. M. , Rahman, R. , Islam, S. M. R. , & Khan, M. S. (2017). A review on mechanisms and commercial aspects of food preservation and processing. Agriculture & Food Security, 6(1), 51. 10.1186/s40066-017-0130-8

[fsn33529-bib-0010] Anwar, F. , Abbas, A. , Mehmood, T. , Gilani, A. H. , & Rehman, N. (2019). *Mentha*: A genus rich in vital nutra‐pharmaceuticals—A review. Phytotherapy Research, 33(10), 2548–2570. 10.1002/ptr.6423 31286590

[fsn33529-bib-0011] Anwar, F. , Ali, M. , Hussain, A. I. , & Shahid, M. (2009). Antioxidant and antimicrobial activities of essential oil and extracts of fennel (*Foeniculum vulgare* mill.) seeds from Pakistan. Flavour and Fragrance Journal, 24(4), 170–176. 10.1002/ffj.1929

[fsn33529-bib-0012] Assami, K. , Pingret, D. , Chemat, S. , Meklati, B. Y. , & Chemat, F. (2012). Ultrasound induced intensification and selective extraction of essential oil from *Carum carvi* L. seeds. Chemical Engineering and Processing: Process Intensification, 62, 99–105. 10.1016/j.cep.2012.09.003

[fsn33529-bib-0013] Ateeq‐Ur‐Rehman , Mannan, A. , Inayatullah, S. , Akhtar, M. Z. , Qayyum, M. , & Mirza, B. (2009). Biological evaluation of wild thyme (*Thymus serpyllum*). Pharmaceutical Biology, 47(7), 628–633. 10.1080/13880200902915622

[fsn33529-bib-0014] Avato, P. , Tursi, F. , Vitali, C. , Miccolis, V. , & Candido, V. (2000). Allylsulfide constituents of garlic volatile oil as antimicrobial agents. Phytomedicine, 7(3), 239–243. 10.1016/S0944-7113(00)80010-0 11185736

[fsn33529-bib-0015] Badgujar, S. B. , Patel, V. V. , & Bandivdekar, A. H. (2014). *Foeniculum vulgare* mill: A review of its botany, phytochemistry, pharmacology, contemporary application, and toxicology. BioMed Research International, 2014, 1–32. 10.1155/2014/842674 PMC4137549 25162032

[fsn33529-bib-0016] Badral, D. , Odonbayar, B. , Murata, T. , Munkhjargal, T. , Tuvshintulga, B. , Igarashi, I. , Suganuma, K. , Inoue, N. , Brantner, A. H. , Odontuya, G. , Sasaki, K. , & Batkhuu, J. (2017). Flavonoid and galloyl glycosides isolated from *Saxifraga spinulosa* and their antioxidative and inhibitory activities against species that cause piroplasmosis. Journal of Natural Products, 80(9), 2416–2423. 10.1021/acs.jnatprod.7b00142 28832147

[fsn33529-bib-0017] Bag, A. , & Chattopadhyay, R. R. (2015). Evaluation of synergistic antibacterial and antioxidant efficacy of essential oils of spices and herbs in combination. PLoS One, 10(7), e0131321. 10.1371/journal.pone.0131321 26132146 PMC4488432

[fsn33529-bib-0018] Bajpai, V. K. , Kamle, M. , Shukla, S. , Mahato, D. K. , Chandra, P. , Hwang, S. K. , Kumar, P. , Huh, Y. S. , & Han, Y. K. (2018). Prospects of using nanotechnology for food preservation, safety, and security. Journal of Food and Drug Analysis, 26(4), 1201–1214. 10.1016/j.jfda.2018.06.011 30249319 PMC9298566

[fsn33529-bib-0019] Bakkali, F. , Averbeck, S. , Averbeck, D. , & Idaomar, M. (2008). Biological effects of essential oils–a review. Food and Chemical Toxicology, 46(2), 446–475. 10.1016/j.fct.2007.09.106 17996351

[fsn33529-bib-0020] Banerjee, G. , & Mukherjee, A. (2002). Biological activity of a common weed–*Portulaca oleracea* L. II. Antifungal activity. Acta Botanica Hungarica, 44(3–4), 205–208. 10.1556/ABot.44.2002.3-4.1

[fsn33529-bib-0021] Barros, L. , Carvalho, A. M. , & Ferreira, I. C. F. R. (2010). Leaves, flowers, immature fruits and leafy flowered stems of *Malva sylvestris*: A comparative study of the nutraceutical potential and composition. Food and Chemical Toxicology, 48(6), 1466–1472. 10.1016/j.fct.2010.03.012 20233600

[fsn33529-bib-0022] Batiha, G. E. S. , Beshbishy, A. M. , Wasef, L. G. , Elewa, Y. H. A. , Al‐Sagan, A. A. , El‐Hack, M. E. A. , Taha, A. E. , Abd‐Elhakim, Y. M. , & Devkota, H. P. (2020). Chemical constituents and pharmacological activities of garlic (*Allium sativum* L.): A review. Nutrients, 12(3), 1–21. 10.3390/nu12030872 PMC7146530 32213941

[fsn33529-bib-0023] Belabdelli, F. , Piras, A. , Bekhti, N. , Falconieri, D. , Belmokhtar, Z. , & Merad, Y. (2020). Chemical composition and antifungal activity of *Foeniculum vulgare* mill. Chemistry Africa, 3(2), 323–328. 10.1007/s42250-020-00130-x

[fsn33529-bib-0024] Ben Abdesslem, S. , Boulares, M. , Elbaz, M. , Ben Moussa, O. , St‐Gelais, A. , Hassouna, M. , & Aider, M. (2021). Chemical composition and biological activities of fennel (*Foeniculum vulgare* mill.) essential oils and ethanolic extracts of conventional and organic seeds. Journal of Food Processing and Preservation, 45(1), 15034. 10.1111/jfpp.15034

[fsn33529-bib-0025] Benabdallah, A. , Rahmoune, C. , Boumendjel, M. , Aissi, O. , & Messaoud, C. (2016). Total phenolic content and antioxidant activity of six wild *Mentha* species (Lamiaceae) from northeast of Algeria. Asian Pacific Journal of Tropical Biomedicine, 6(9), 760–766. 10.1016/j.apjtb.2016.06.016

[fsn33529-bib-0026] Bhargava, K. , Conti, D. S. , da Rocha, S. R. P. , & Zhang, Y. (2015). Application of an oregano oil nanoemulsion to the control of foodborne bacteria on fresh lettuce. Food Microbiology, 47, 69–73. 10.1016/j.fm.2014.11.007 25583339

[fsn33529-bib-0027] Birasuren, B. , Oh, H. L. , Kim, C. R. , Kim, N. Y. , Jeon, H. L. , & Kim, M. R. (2012). Antioxidant activities of *Ribes diacanthum* pall extracts in the northern region of Mongolia. Preventive Nutrition and Food Science, 17(4), 261–268. 10.3746/pnf.2012.17.4.261 24471094 PMC3866731

[fsn33529-bib-0028] Biswas, N. N. , Saha, S. , & Ali, M. K. (2014). Antioxidant, antimicrobial, cytotoxic and analgesic activities of ethanolic extract of *Mentha arvensis* L. Asian Pacific Journal of Tropical Biomedicine, 4(10), 792–797. 10.12980/APJTB.4.2014C1298

[fsn33529-bib-0029] Bogavac, M. , Karaman, M. , Janjušević, L. , Sudji, J. , Radovanović, B. , Novaković, Z. , Simeunović, J. , & Božin, B. (2015). Alternative treatment of vaginal infections–*in vitro* antimicrobial and toxic effects of *Coriandrum sativum* L. and *Thymus vulgaris* L. essential oils. Journal of Applied Microbiology, 119(3), 697–710. 10.1111/jam.12883 26109513

[fsn33529-bib-0030] Bokhari, N. , Perveen, K. , Al Khulaifi, M. , Kumar, A. , & Siddiqui, I. (2016). *In vitro* antibacterial activity and chemical composition of essential oil of *Mentha arvensis* Linn. Leaves. Journal of Essential Oil‐Bearing Plants, 19(4), 907–915. 10.1080/0972060X.2016.1184993

[fsn33529-bib-0031] Brewer, M. S. (2011). Natural antioxidants: Sources, compounds, mechanisms of action, and potential applications. Comprehensive Reviews in Food Science and Food Safety, 10(4), 221–247. 10.1111/j.1541-4337.2011.00156.x

[fsn33529-bib-0032] Buyankhishig, B. , Murata, T. , Odonbayar, B. , Batkhuu, J. , & Sasaki, K. (2021). New compounds from the aerial parts of *Calligonum mongolicum* . Phytochemistry Letters, 41(July 2020), 147–151. 10.1016/j.phytol.2020.12.002

[fsn33529-bib-0033] Calo, J. R. , Crandall, P. G. , O'Bryan, C. A. , & Ricke, S. C. (2015). Essential oils as antimicrobials in food systems–A review. Food Control, 54, 111–119. 10.1016/j.foodcont.2014.12.040

[fsn33529-bib-0034] Campêlo, M. C. S. , Medeiros, J. M. S. , & Silva, J. B. A. (2019). Natural products in food preservation. International Food Research Journal, 26(1), 41–46.

[fsn33529-bib-0035] Carocho, M. , Barreiro, M. F. , Morales, P. , & Ferreira, I. C. F. R. (2014). Adding molecules to food, pros and cons: A review on synthetic and natural food additives. Comprehensive Reviews in Food Science and Food Safety, 13(4), 377–399. 10.1111/1541-4337.12065 33412697

[fsn33529-bib-0036] Carocho, M. , Morales, P. , & Ferreira, I. C. F. R. (2015). Natural food additives: *Quo vadis*? Trends in Food Science and Technology, 45(2), 284–295. 10.1016/j.tifs.2015.06.007

[fsn33529-bib-0037] Cecotti, R. , Bergomi, P. , Carpana, E. , & Tava, A. (2016). Chemical characterization of the volatiles of leaves and flowers from cultivated *Malva sylvestris* var. *mauritiana* and their antimicrobial activity against the aetiological agents of the European and American foulbrood of honeybees (*Apis mellifera*). Natural Product Communications, 11(10), 1527–1530. 10.1177/1934578x1601101026 30549614

[fsn33529-bib-0038] Cheema, H. S. , Prakash, O. , Pal, A. , Khan, F. , Bawankule, D. U. , & Darokar, M. P. (2014). Glabridin induces oxidative stress mediated apoptosis like cell death of malaria parasite *Plasmodium falciparum* . Parasitology International, 63(2), 349–358. 10.1016/j.parint.2013.12.005 24361284

[fsn33529-bib-0039] Chen, Y. , Ding, Z. , Wu, Y. , Chen, Q. , Liu, M. , Yu, H. , Wang, D. , Zhang, Y. , & Wang, T. (2020). Effects of *Allium mongolicum* regel and its flavonoids on constipation. Biomolecules, 10(1), 14. 10.3390/biom10010014 PMC7022811 31877639

[fsn33529-bib-0040] Cheng, C. , & Wang, Z. (2006). Bacteriostasic activity of anthocyanin of *Malva sylvestris* . Journal of Forestry Research, 17(1), 83–85. 10.1007/s11676-006-0020-6

[fsn33529-bib-0041] Chhatre, S. , Nesari, T. , Somani, G. , Kanchan, D. , & Sathaye, S. (2014). Phytopharmacological overview of *Tribulus terrestris* . Pharmacognosy Reviews, 8(15), 45–51. 10.4103/0973-7847.125530 24600195 PMC3931200

[fsn33529-bib-0042] Chishti, S. , Kaloo, Z. A. , & Sultan, P. (2013). Medicinal importance of genus *Origanum*: A review. Journal of Pharmacognosy and Phytotherapy, 5(10), 170–177. 10.5897/JPP2013.0285

[fsn33529-bib-0043] Choi, E. M. , & Hwang, J. K. (2004). Antiinflammatory, analgesic and antioxidant activities of the fruit of *Foeniculum vulgare* . Fitoterapia, 75(6), 557–565. 10.1016/j.fitote.2004.05.005 15351109

[fsn33529-bib-0044] Chouitah, O. , Meddah, B. , Aoues, A. , & Sonnet, P. (2011). Chemical composition and antimicrobial activities of the essential oil from *Glycyrrhiza glabra* leaves. Journal of Essential Oil‐Bearing Plants, 14(3), 284–288. 10.1080/0972060X.2011.10643935

[fsn33529-bib-0045] Conforti, F. , Statti, G. , Uzunov, D. , & Menichini, F. (2006). Comparative chemical composition and antioxidant activities of wild and cultivated *Laurus nobilis* L. leaves and *Foeniculum vulgare* subsp. *piperitum* (Ucria) coutinho seeds. Biological and Pharmaceutical Bulletin, 29(10), 2056–2064. 10.1248/bpb.29.2056 17015951

[fsn33529-bib-0046] Dar, S. A. , Ganai, F. A. , Yousuf, A. R. , Balkhi, M. U. H. , Bhat, T. M. , & Sharma, P. (2013). Pharmacological and toxicological evaluation of *Urtica dioica* . Pharmaceutical Biology, 51(2), 170–180. 10.3109/13880209.2012.715172 23036051

[fsn33529-bib-0047] Dauqan, E. M. A. , & Abdullah, A. (2017). Medicinal and functional values of thyme (*Thymus vulgaris* L.) herb. Journal of Applied Biology & Biotechnology, 5(2), 17–22. 10.7324/jabb.2017.50203

[fsn33529-bib-0048] De Falco, E. , Mancini, E. , Roscigno, G. , Mignola, E. , Taglialatela‐Scafati, O. , & Senatore, F. (2013). Chemical composition and biological activity of essential oils of *Origanum vulgare* L. subsp. *vulgare* L. under different growth conditions. Molecules, 18(12), 14948–14960. 10.3390/molecules181214948 24304588 PMC6270476

[fsn33529-bib-0049] De Martino, L. , De Feo, V. , Formisano, C. , Mignola, E. , & Senatore, F. (2009). Chemical composition and antimicrobial activity of the essential oils from three chemotypes of *Origanum vulgare* L ssp. *hirtum* (Link) Ietswaart growing wild in Campania (Southern Italy). Molecules, 14(8), 2735–2746. 10.3390/molecules14082735 19701120 PMC6254797

[fsn33529-bib-0050] Delaquis, P. J. , Stanich, K. , Girard, B. , & Mazza, G. (2002). Antimicrobial activity of individual and mixed fractions of dill, cilantro, coriander and eucalyptus essential oils. International Journal of Food Microbiology, 74(1–2), 101–109. 10.1016/S0168-1605(01)00734-6 11929164

[fsn33529-bib-0051] Dharmananda, S. (2004). Oldenlandia and Scutellaria antitoxin and anticancer herbs . http://www.itmonline.org/arts/oldenlandia.htm

[fsn33529-bib-0052] Diana, M. , Sales, C. , Costa, H. B. , & Meira, D. D. (2016). Ameliorative properties of Iranian *Trigonella foenum‐graecum* L. seeds and *Punica granatum* L. peel extracts in streptozotocin‐induced experimental diabetic Guinea pigs. Asian Pacific Journal of Tropical Biomedicine, 6(1), 26–31.

[fsn33529-bib-0053] Diao, W. R. , Hu, Q. P. , Zhang, H. , & Xu, J. G. (2014). Chemical composition, antibacterial activity and mechanism of action of essential oil from seeds of fennel (*Foeniculum vulgare* mill.). Food Control, 35(1), 109–116. 10.1016/j.foodcont.2013.06.056

[fsn33529-bib-0054] Ding, H. , Liu, W. , Erdene, K. , Du, H. , & Ao, C. (2021). Effects of dietary supplementation with *Allium mongolicum* regel extracts on growth performance, serum metabolites, immune responses, antioxidant status, and meat quality of lambs. Animal Nutrition, 7(2), 530–538. 10.1016/j.aninu.2021.04.001 34258442 PMC8245812

[fsn33529-bib-0055] Dong, Y. , Ruan, J. , Ding, Z. , Zhao, W. , Hao, M. , Zhang, Y. , Jiang, H. , Zhang, Y. , & Wang, T. (2020). Phytochemistry and comprehensive chemical profiling study of flavonoids and phenolic acids in the aerial parts of *Allium mongolicum* regel and their intestinal motility evaluation. Molecules, 25(3), 577. 10.3390/molecules25030577 32013151 PMC7036834

[fsn33529-bib-0056] Dorman, H. J. D. , Koşar, M. , Kahlos, K. , Holm, Y. , & Hiltunen, R. (2003). Antioxidant properties and composition of aqueous extracts from *Mentha* species, hybrids, varieties, and cultivars. Journal of Agricultural and Food Chemistry, 51(16), 4563–4569. 10.1021/jf034108k 14705878

[fsn33529-bib-0057] Dua, A. , Agrawal, S. , Kaur, A. , & Mahajan, R. (2014). Antioxidant profile of *Coriandrum sativum* methanolic extract. International Research Journal of Pharmacy, 5(3), 220–224. 10.7897/2230-8407.050347

[fsn33529-bib-0058] Dulger, B. , Goncu, B. S. , & Gucin, F. (2010). Antibacterial activity of the seeds of *Hyoscyamus Niger* L. (henbane). Asian Journal of Chemistry, 22(9), 6879–6883.

[fsn33529-bib-0059] El Babili, F. , Bouajila, J. , Souchard, J. P. , Bertrand, C. , Bellvert, F. , Fouraste, I. , Moulis, C. , & Valentin, A. (2011). Oregano: Chemical analysis and evaluation of its antimalarial, antioxidant, and cytotoxic activities. Journal of Food Science, 76(3), C512–C518. 10.1111/j.1750-3841.2011.02109.x 21535822

[fsn33529-bib-0060] El‐Sayed, S. M. , & Youssef, A. M. (2019). Potential application of herbs and spices and their effects in functional dairy products. Heliyon, 5(6), e01989. 10.1016/j.heliyon.2019.e01989 31338458 PMC6607025

[fsn33529-bib-0061] Engels, C. , Schieber, A. , & Gänzle, M. G. (2012). Sinapic acid derivatives in defatted oriental mustard (*Brassica juncea* L.) seed meal extracts using UHPLC‐DAD‐ESI‐MS^n^ and identification of compounds with antibacterial activity. European Food Research and Technology, 234(3), 535–542. 10.1007/s00217-012-1669-z

[fsn33529-bib-0062] Enkhtuya, E. , Kashiwagi, T. , Shimamura, T. , Ukeda, H. , & Tseye‐Oidov, O. (2014). Screening study on antioxidant activity of plants grown wildly in Mongolia. Food Science and Technology Research, 20(4), 891–897. 10.3136/fstr.20.891

[fsn33529-bib-0063] Enkhtuya, E. , Shimamura, T. , Kashiwagi, T. , & Ukeda, H. (2017). Antioxidative constituents in the leaves of *Paeonia anomala* grown in Mongolia. Food Science and Technology Research, 23(1), 63–70. 10.3136/fstr.23.63

[fsn33529-bib-0064] Erdenetsogt, U. , Gotov, C. , Voigt, K. , Bartram, S. , Boland, W. , & Dagvadorj, E. (2019). Chemical composition and antimicrobial activity of essential oil from *pyrethrum pulchrum* Ledeb. Journal of Pharmacognosy & Natural Products, 4(2), 38–43. 10.4172/2472-0992.1000154

[fsn33529-bib-0065] FAO . (2011). Global food losses and food waste – Extent, causes and prevention . https://www.fao.org/3/i2697e/i2697e.pdf

[fsn33529-bib-0066] FAO . (2017). The state of food security and nutrition in Europe and central Asia . http://www.fao.org/3/a‐i8194e.pdf

[fsn33529-bib-0067] Farid, A. , Kamel, D. , Abdelwahab Montaser, S. , Mohamed Ahmed, M. , El Amir, M. , & El Amir, A. (2020). Assessment of antioxidant, immune enhancement, and antimutagenic efficacy of fennel seed extracts in irradiated human blood cultures. Journal of Radiation Research and Applied Sciences, 13(1), 260–266. 10.1080/16878507.2020.1728963

[fsn33529-bib-0068] Feizi, S. , Taghipour, E. , Ghadam, P. , & Mohammadi, P. (2018). Antifungal, antibacterial, antibiofilm and colorimetric sensing of toxic metals activities of eco friendly, economical synthesized Ag/AgCl nanoparticles using *Malva Sylvestris* leaf extracts. Microbial Pathogenesis, 125, 33–42. 10.1016/j.micpath.2018.08.054 30171981

[fsn33529-bib-0069] Fernández‐Poyatos, M. D. P. , Llorent‐Martínez, E. J. , & Ruiz‐Medina, A. (2021). Phytochemical composition and antioxidant activity of *Portulaca oleracea*: Influence of the steaming cooking process. Food, 10(1), 94. 10.3390/foods10010094 PMC7824898 33466382

[fsn33529-bib-0070] Fliou, J. , Riffi, O. , Amechrouq, A. , Elhourri, M. , & Ghouati, Y. (2020). Comparative study of the chemical composition of the essential oil of *Origanum compactum* from the seven regions of Morocco and their antimicrobial activity. Journal of Microbiology, Biotechnology and Food Sciences, 10(1), 42–48. 10.15414/jmbfs.2020.10.1.42-48

[fsn33529-bib-0071] Fredotovíc, Ž. , Šprung, M. , Soldo, B. , Ljubenkov, I. , Budić‐Leto, I. , Bilušić, T. , Cikeš‐Čulić, V. , & Puizina, J. (2017). Chemical composition and biological activity of *Allium cepa* L. and *allium × cornutum* (Clementi ex Visiani 1842) methanolic extracts. Molecules, 22(3), 448. 10.3390/molecules22030448 28287477 PMC6155300

[fsn33529-bib-0072] Freires, I. D. A. , Murata, R. M. , Furletti, V. F. , Sartoratto, A. , De Alencar, S. M. , Figueira, G. M. , Rodrigues, J. A. D. O. , Duarte, M. C. T. , & Rosalen, P. L. (2014). *Coriandrum sativum* L. (coriander) essential oil: Antifungal activity and mode of action on *Candida* spp., and molecular targets affected in human whole‐genome expression. PLoS One, 9(6), e99086. 10.1371/journal.pone.0099086 24901768 PMC4047076

[fsn33529-bib-0073] Frisvad, J. C. , Hubka, V. , Ezekiel, C. N. , Hong, S. B. , Nováková, A. , Chen, A. J. , Arzanlou, M. , Larsen, T. O. , Sklenář, F. , Mahakarnchanakul, W. , Samson, R. A. , & Houbraken, J. (2019). Taxonomy of *aspergillus* section *Flavi* and their production of aflatoxins, ochratoxins and other mycotoxins. Studies in Mycology, 93, 1–63. 10.1016/j.simyco.2018.06.001 30108412 PMC6080641

[fsn33529-bib-0074] Galovičová, L. , Borotová, P. , Valková, V. , Vukovic, N. L. , Vukic, M. , Štefániková, J. , Ďúranová, H. , Kowalczewski, P. Ł. , Čmiková, N. , & Kačániová, M. (2021). *Thymus vulgaris* essential oil and its biological activity. Plants, 10(9), 1–17. 10.3390/plants10091959 PMC8467294 34579491

[fsn33529-bib-0075] Gao, J. , Yin, W. , & Corcoran, O. (2019). From *Scutellaria barbata* to BZL101 in cancer patients: Phytochemistry, pharmacology, and clinical evidence. Natural Product Communications, 14(10), 1–12. 10.1177/1934578X19880645

[fsn33529-bib-0076] Gatea, F. , Dumitra Teodor, E. , Maria Seciu, A. , Nagodă, E. , & Lucian Radu, G. (2017). Chemical constituents and bioactive potential of *Portulaca pilosa* L vs *Portulaca Oleracea* L. Medicinal Chemistry Research, 26(7), 1516–1527. 10.1007/s00044-017-1862-5

[fsn33529-bib-0077] Gendaram, O. , Choi, Y. H. , Kim, Y. S. , & Ryu, S. Y. (2011). Anti‐oxidative and antibacterial constituents from *Sedum hybridum* . Natural Product Sciences, 17(4), 279–284.

[fsn33529-bib-0078] Gharras, H. E. (2009). Polyphenols : Food sources, properties and applications – A review. International Journal of Food Science & Technology, 44, 2512–2518. 10.1111/j.1365-2621.2009.02077.x

[fsn33529-bib-0079] Giordani, C. , Simonetti, G. , Natsagdorj, D. , Choijamts, G. , Ghirga, F. , Calcaterra, A. , Quaglio, D. , De Angelis, G. , Toniolo, C. , & Pasqua, G. (2020). Antifungal activity of Mongolian medicinal plant extracts. Natural Product Research, 34(4), 449–455. 10.1080/14786419.2019.1610960 31135192

[fsn33529-bib-0080] Gonchig, E. , Erdenebat, S. , Togtoo, O. , Bataa, S. , Gendaram, O. , Young, S. K. , & Shi, Y. R. (2008). Antimicrobial activity of Mongolian medicinal plants. Natural Product Sciences, 14(1), 32–36 https://www.koreascience.or.kr/article/JAKO200817663908980.pdf

[fsn33529-bib-0081] Goswami, N. , & Chatterjee, S. (2014). Assessment of free radical scavenging potential and oxidative DNA damage preventive activity of *Trachyspermum ammi* L. (carom) and *Foeniculum vulgare* mill. (fennel) seed extracts. BioMed Research International, 2014, 1–8. 10.1155/2014/582767 PMC4130144 25143939

[fsn33529-bib-0082] Grauso, L. , de Falco, B. , Lanzotti, V. , & Motti, R. (2020). Stinging nettle, *Urtica dioica* L.: Botanical, phytochemical and pharmacological overview. Phytochemistry Reviews, 19(6), 1341–1377. 10.1007/s11101-020-09680-x

[fsn33529-bib-0083] Gupta, V. K. , Fatima, A. , Faridi, U. , Negi, A. S. , Shanker, K. , Kumar, J. K. , Rahuja, N. , Luqman, S. , Sisodia, B. S. , Saikia, D. , Darokar, M. P. , & Khanuja, S. P. S. (2008). Antimicrobial potential of *Glycyrrhiza glabra* roots. Journal of Ethnopharmacology, 116(2), 377–380. 10.1016/j.jep.2007.11.037 18182260

[fsn33529-bib-0084] Gutiérrez‐del‐Río, I. , Fernández, J. , & Lombó, F. (2018). Plant nutraceuticals as antimicrobial agents in food preservation: Terpenoids, polyphenols and thiols. International Journal of Antimicrobial Agents, 52(3), 309–315. 10.1016/j.ijantimicag.2018.04.024 29777759

[fsn33529-bib-0085] Hajlaoui, H. , Arraouadi, S. , Noumi, E. , Aouadi, K. , Adnan, M. , Khan, M. A. , Kadri, A. , & Snoussi, M. (2021). Antimicrobial, antioxidant, anti‐acetylcholinesterase, antidiabetic, and pharmacokinetic properties of *Carum carvi* L. and *Coriandrum sativum* L. essential oils alone and in combination. Molecules, 26(12), 3625. 10.3390/molecules26123625 34199316 PMC8231812

[fsn33529-bib-0086] He, W. , & Huang, B. (2011). A review of chemistry and bioactivities of a medicinal spice: *Foeniculum vulgare* . Journal of Medicinal Plants Research, 5(16), 3595–3600 http://www.academicjournals.org/JMPR

[fsn33529-bib-0087] Hernández‐Hernández, E. , Regalado‐González, C. , Vázquez‐Landaverde, P. , Guerrero‐Legarreta, I. , & García‐Almendárez, B. E. (2014). Microencapsulation, chemical characterization, and antimicrobial activity of Mexican (*Lippia graveolens* H.B.K.) and European (*Origanum vulgare* L.) oregano essential oils. Scientific World Journal, 2014, 1–12. 10.1155/2014/641814 PMC4142163 25177730

[fsn33529-bib-0088] Heydari, M. , Zanfardino, A. , Taleei, A. , Bushehri, A. A. S. , Hadian, J. , Maresca, V. , Sorbo, S. , Di Napoli, M. , Varcamonti, M. , Basile, A. , & Rigano, D. (2018). Effect of heat stress on yield, monoterpene content and antibacterial activity of essential oils of *Mentha x piperita* var. Mitcham and *Mentha arvensis* var. *piperascens* . Molecules, 23(8), 1903. 10.3390/molecules23081903 30061551 PMC6222296

[fsn33529-bib-0089] Höferl, M. , Wanner, J. , Tabanca, N. , Ali, A. , Gochev, V. , Schmidt, E. , Kaul, V. K. , Singh, V. , & Jirovetz, L. (2020). Biological activity of *Matricaria chamomilla* essential oils of various chemotypes. Planta Medica International Open, 7(3), e114–e121. 10.1055/a-1186-2400

[fsn33529-bib-0090] Hosseinzadeh, H. , & Nassiri‐Asl, M. (2015). Pharmacological effects of *Glycyrrhiza* spp. and its bioactive constituents: Update and review. Phytotherapy Research, 29(12), 1868–1886. 10.1002/ptr.5487 26462981

[fsn33529-bib-0091] Hromiš, N. M. , Lazić, V. L. , Markov, S. L. , Vaštag, Ž. G. , Popović, S. Z. , Šuput, D. Z. , Džinić, N. R. , Velićanski, A. S. , & Popović, L. M. (2015). Optimization of chitosan biofilm properties by addition of caraway essential oil and beeswax. Journal of Food Engineering, 158, 86–93. 10.1016/j.jfoodeng.2015.01.001

[fsn33529-bib-0092] Hussain, A. I. , Anwar, F. , Nigam, P. S. , Ashraf, M. , & Gilani, A. H. (2010). Seasonal variation in content, chemical composition and antimicrobial and cytotoxic activities of essential oils from four *Mentha* species. Journal of the Science of Food and Agriculture, 90(11), 1827–1836. 10.1002/jsfa.4021 20602517

[fsn33529-bib-0093] Irani, M. , Sarmadi, M. , Bernard, F. , Ebrahimi Pour, G. H. , & Bazarnov, H. S. (2010). Leaves antimicrobial activity of *Glycyrrhiza glabra* L. Iranian Journal of Pharmaceutical Research, 9(4), 425–428. 10.22037/ijpr.2010.909 24381608 PMC3870067

[fsn33529-bib-0094] Iranshahy, M. , Javadi, B. , Iranshahi, M. , Jahanbakhsh, S. P. , Mahyari, S. , Hassani, F. V. , & Karimi, G. (2017). A review of traditional uses, phytochemistry and pharmacology of *Portulaca oleracea* L. Journal of Ethnopharmacology, 205, 158–172. 10.1016/j.jep.2017.05.004 28495602

[fsn33529-bib-0095] Iseppi, R. , Sabia, C. , de Niederhäusern, S. , Pellati, F. , Benvenuti, S. , Tardugno, R. , Bondi, M. , & Messi, P. (2019). Antibacterial activity of *Rosmarinus officinalis* L. and *Thymus vulgaris* L. essential oils and their combination against food‐borne pathogens and spoilage bacteria in ready‐to‐eat vegetables. Natural Product Research, 33(24), 3568–3572. 10.1080/14786419.2018.1482894 29874951

[fsn33529-bib-0096] Iseppi, R. , Tardugno, R. , Brighenti, V. , Benvenuti, S. , Sabia, C. , Pellati, F. , & Messi, P. (2020). Phytochemical composition and *in vitro* antimicrobial activity of essential oils from the *Lamiaceae* family against *Streptococcus agalactiae* and *Candida albicans* biofilms. Antibiotics, 9(9), 1–16. 10.3390/antibiotics9090592 PMC7558348 32927692

[fsn33529-bib-0097] Jang, M. H. , Piao, X. L. , Kim, J. M. , Kwon, S. W. , & Park, J. H. (2008). Inhibition of cholinesterase and amyloid‐β aggregation by resveratrol oligomers from *Vitis amurensis* . Phytotherapy Research, 22(4), 544–549. 10.1002/ptr 18338769

[fsn33529-bib-0098] Jeršek, B. , Ulrih, N. P. , Skrt, M. , Gavarić, N. , Božin, B. , & Možina, S. S. (2014). Effects of selected essential oils on the growth and production of ochratoxin A by *Penicillium verrucosum* . Archives of Industrial Hygiene and Toxicology, 65(2), 199–208. 10.2478/10004-1254-65-2014-2486 24945417

[fsn33529-bib-0099] Jianu, C. , Stoin, D. , Cocan, I. , David, I. , Pop, G. , Lukinich‐gruia, A. T. A. T. A. T. , Mioc, M. , Mioc, A. , Șoica, C. , Muntean, D. , Rusu, L.‐C. , Goleț, I. , Horhat, D. I. , Potential, A. , Graham, R. , Jianu, C. , Stoin, D. , Cocan, I. , David, I. , … Horhat, D. I. (2021). In silico and *in vitro* evaluation of the antimicrobial and antioxidant potential of *Mentha × smithiana* R GRAHAM essential oil from Western Romania. Foods, 10(4), 815. 10.3390/foods10040815 33918674 PMC8069324

[fsn33529-bib-0100] Kačániová, M. , Galovičová, L. , Ivanišová, E. , Vukovic, N. L. , Štefániková, J. , Valková, V. , Borotová, P. , Žiarovská, J. , Terentjeva, M. , Felšöciová, S. , & Tvrdá, E. (2020). Antioxidant, antimicrobial and antibiofilm activity of coriander (*Coriandrum sativum* L.) essential oil for its application in foods. Food, 9(3), 282–301. 10.3390/foods9030282 PMC7142854 32143314

[fsn33529-bib-0101] Kalemba, D. , & Synowiec, A. (2020). Agrobiological interactions of essential oils of two menthol mints: *Mentha piperita* and *Mentha arvensis* . Molecules, 25(1), 1–33. 10.3390/molecules25010059 PMC6983130 31878007

[fsn33529-bib-0102] Karahan, F. , Avsar, C. , Ozyigit, I. I. , & Berber, I. (2016). Antimicrobial and antioxidant activities of medicinal plant *Glycyrrhiza glabra* var. *glandulifera* from different habitats. Biotechnology and Biotechnological Equipment, 30(4), 797–804. 10.1080/13102818.2016.1179590

[fsn33529-bib-0103] Karkanis, A. , Martins, N. , Petropoulos, S. A. , & Ferreira, I. C. F. R. (2018). Phytochemical composition, health effects, and crop management of liquorice (*Glycyrrhiza glabra* L.): Α medicinal plant. Food Reviews International, 34(2), 182–203. 10.1080/87559129.2016.1261300

[fsn33529-bib-0104] Kaur, G. J. , & Arora, D. S. (2009). Antibacterial and phytochemical screening of *Anethum graveolens*, *Foeniculum vulgare* and *Trachyspermum ammi* . BMC Complementary and Alternative Medicine, 9, 1–10. 10.1186/1472-6882-9-30 19656417 PMC2736926

[fsn33529-bib-0246] Каримов, А. М. , & Ботиров, Э. Х. (2016). Структурное разнообразие и степень изученности флавоноидов рода Scutellaria L. Химия растительного сырья, (1), 5‐28. 10.14258/jcprm.201601962

[fsn33529-bib-0105] Kazemi, M. (2015). Chemical composition and antimicrobial activity of essential oil of *Matricaria recutita* . International Journal of Food Properties, 18(8), 1784–1792. 10.1080/10942912.2014.939660

[fsn33529-bib-0106] Khan, A. A. , Amjad, M. S. , & Saboon . (2019). GC‐MS analysis and biological activities of *Thymus vulgaris* and *Mentha arvensis* essential oil. Turkish Journal of Biochemistry, 44(3), 388–396. 10.1515/tjb-2018-0258

[fsn33529-bib-0107] Khan, S. , Fatima, I. , Kazmi, M. H. , & Malik, A. (2015). A new steroidal alkaloid from *Allium victorialis* . Chemistry of Natural Compounds, 51(6), 1134–1137. 10.1007/s10600-015-1509-z

[fsn33529-bib-0108] Kim, J. , Joo, I. , Kim, H. , & Han, Y. (2013). 18β‐Glycyrrhetinic acid induces immunological adjuvant activity of Th1 against *Candida albicans* surface mannan extract. Phytomedicine, 20(11), 951–955. 10.1016/j.phymed.2013.04.008 23746951

[fsn33529-bib-0109] Kocić‐Tanackov, S. , Dimić, G. , Đerić, N. , Mojović, L. , Tomović, V. , Šojić, B. , Đukić‐Vuković, A. , & Pejin, J. (2020). Growth control of molds isolated from smoked fermented sausages using basil and caraway essential oils, *in vitro* and *in vivo* . LWT–Food Science and Technology, 123, 109095. 10.1016/j.lwt.2020.109095

[fsn33529-bib-0110] Kocić‐Tanackov, S. , Dimić, G. , Mojović, L. , Gvozdanović‐Varga, J. , Djukić‐Vuković, A. , Tomović, V. , Šojić, B. , & Pejin, J. (2017). Antifungal activity of the onion (*Allium cepa* L.) essential oil against *aspergillus*, *Fusarium* and *Penicillium* species isolated from food. Journal of Food Processing and Preservation, 41(4), e13050. 10.1111/jfpp.13050

[fsn33529-bib-0111] Koldaş, S. , Demirtas, I. , Ozen, T. , Demirci, M. A. , & Behçet, L. (2015). Phytochemical screening, anticancer and antioxidant activities of *Origanum vulgare* L. ssp. *viride* (Boiss.) Hayek, a plant of traditional usage. Journal of the Science of Food and Agriculture, 95(4), 786–798. 10.1002/jsfa.6903 25200133

[fsn33529-bib-0112] Kordali, S. , Cakir, A. , Mavi, A. , Kilic, H. , & Yildirim, A. (2005). Screening of chemical composition and antifungal and antioxidant activities of the essential oils from three Turkish *Artemisia* species. Journal of Agricultural and Food Chemistry, 53(5), 1408–1416. 10.1021/jf048429n 15740015

[fsn33529-bib-0113] Kurilov, D. V. , Kirichenko, E. B. , Bidyukova, G. F. , Olekhnovich, L. S. , & Ku, L. D. (2009). Composition of the essential oil of introduced mint forms of *Mentha piperita* and *Mentha arvensis* species. Doklady Biological Sciences, 429(1), 538–540. 10.1134/S0012496609060167 20170067

[fsn33529-bib-0114] Kurt, Ş. , Güneş, U. , & Soylu, E. M. (2011). *In vitro* and *in vivo* antifungal activity of synthetic pure isothiocyanates against *Sclerotinia sclerotiorum* . Pest Management Science, 67(7), 869–875. 10.1002/ps.2126 21370393

[fsn33529-bib-0115] Kyung, K. H. (2012). Antimicrobial properties of *allium* species. Current Opinion in Biotechnology, 23(2), 142–147. 10.1016/j.copbio.2011.08.004 21903379

[fsn33529-bib-0116] Lanzotti, V. , Barile, E. , Antignani, V. , Bonanomi, G. , & Scala, F. (2012). Antifungal saponins from bulbs of garlic, *Allium sativum* L. var. *Voghiera* . Phytochemistry, 78, 126–134. 10.1016/j.phytochem.2012.03.009 22513009

[fsn33529-bib-0117] Lanzotti, V. , Romano, A. , Lanzuise, S. , Bonanomi, G. , & Scala, F. (2012). Antifungal saponins from bulbs of white onion, *Allium cepa* L. Phytochemistry, 74, 133–139. 10.1016/j.phytochem.2011.11.008 22169018

[fsn33529-bib-0118] Laribi, B. , Kouki, K. , M'Hamdi, M. , & Bettaieb, T. (2015). Coriander (*Coriandrum sativum* L.) and its bioactive constituents. Fitoterapia, 103, 9–26. 10.1016/j.fitote.2015.03.012 25776008

[fsn33529-bib-0119] Leontiev, R. , Hohaus, N. , Jacob, C. , Gruhlke, M. C. H. , & Slusarenko, A. J. (2018). A comparison of the antibacterial and antifungal activities of thiosulfinate analogues of allicin. Scientific Reports, 8(1), 1–19. 10.1038/s41598-018-25154-9 29712980 PMC5928221

[fsn33529-bib-0120] Li, M. , Zhu, X. , Tian, J. , Liu, M. , & Wang, G. (2019). Dietary flavonoids from *Allium mongolicum* regel promotes growth, improves immune, antioxidant status, immune‐related signaling molecules and disease resistance in juvenile northern snakehead fish (*Channa argus*). Aquaculture, 501, 473–481. 10.1016/j.aquaculture.2018.12.011

[fsn33529-bib-0121] Li, M. Y. , Guo, W. Q. , Guo, G. L. , Zhu, X. M. , Niu, X. T. , Shan, X. F. , Tian, J. X. , Wang, G. Q. , & Zhang, D. M. (2019). Effect of sub‐chronic exposure to selenium and *Allium mongolicum* regel flavonoids on *Channa argus*: Bioaccumulation, oxidative stress, immune responses and immune‐related signaling molecules. Fish and Shellfish Immunology, 91, 122–129. 10.1016/j.fsi.2019.05.002 31055018

[fsn33529-bib-0122] Lombrea, A. , Antal, D. , Ardelean, F. , Avram, S. , Pavel, I. Z. , Vlaia, L. , Mut, A. M. , Diaconeasa, Z. , Dehelean, C. A. , Soica, C. , & Danciu, C. (2020). A recent insight regarding the phytochemistry and bioactivity of *Origanum vulgare* L. essential oil. International Journal of Molecular Sciences, 21(24), 1–28. 10.3390/ijms21249653 PMC7765853 33348921

[fsn33529-bib-0123] Ludwiczuk, A. , Kiełtyka‐Dadasiewicz, A. , Sawicki, R. , Golus, J. , & Ginalska, G. (2016). Essential oils of some *Mentha* species and cultivars, their chemistry and bacteriostatic activity. Natural Product Communications, 11(7), 1015–1018. 10.1177/1934578x1601100736 30452185

[fsn33529-bib-0124] Lukas, B. , Schmiderer, C. , & Novak, J. (2015). Essential oil diversity of European *Origanum vulgare* L. (Lamiaceae). Phytochemistry, 119, 32–40. 10.1016/j.phytochem.2015.09.008 26454793

[fsn33529-bib-0125] Ma, Y. L. , Zhu, D. Y. , Thakur, K. , Wang, C. H. , Wang, H. , Ren, Y. F. , Zhang, J. G. , & Wei, Z. J. (2018). Antioxidant and antibacterial evaluation of polysaccharides sequentially extracted from onion (*Allium cepa* L.). International Journal of Biological Macromolecules, 111, 92–101. 10.1016/j.ijbiomac.2017.12.154 29305215

[fsn33529-bib-0126] Magsar, U. , Nyamsuren, K. , Khadbaatar, S. , Tovuudorj, M. E. , Baasansuren, E. , Indree, T. , Lkhagvadorj, K. , & Kwon, O. (2017). Survey of medicinal plants in the Khuvsgul and Khangai mountain regions of Mongolia. Journal of Ecology and Environment, 41(1), 1–5. 10.1186/s41610-017-0034-3

[fsn33529-bib-0127] Magsar, U. , Vanjil, G. , Tovuudorj, M. , & Baasansuren, E. (2018). Medicinal plant diversity in the southern and eastern Gobi Desert region, Mongolia. Journal of Ecology and Environment, 42(1), 1–13. 10.1186/s41610-018-0064-5

[fsn33529-bib-0128] Majedi, S. , Abdulsattar, T. , Jalal, H. , & Hussain, F. H. S. (2021). A review of biochemical structures of *Urtica dioica* metabolites and their pharmaceutical effects. Chemical Review and Letters, 4, 206–212.

[fsn33529-bib-0129] Malir, F. , Ostry, V. , Pfohl‐Leszkowicz, A. , Malir, J. , & Toman, J. (2016). Ochratoxin A: 50 years of research. Toxins, 8(7), 12–15. 10.3390/toxins8070191 PMC4963825 27384585

[fsn33529-bib-0130] Mandal, S. , & DebMandal, M. (2016). Essential oils in food preservation, flavor and safety (pp. 825–834). Elsevier Inc. 10.1016/B978-0-12-416641-7.00094-8

[fsn33529-bib-0131] Mandal, S. , & Mandal, M. (2015). Coriander (*Coriandrum sativum* L.) essential oil: Chemistry and biological activity. Asian Pacific Journal of Tropical Biomedicine, 5(6), 421–428. 10.1016/j.apjtb.2015.04.001

[fsn33529-bib-0132] Mantle, P. , Kilic, M. A. , Mor, F. , & Ozmen, O. (2015). Contribution of organ vasculature in rat renal analysis for ochratoxin A: Relevance to toxicology of nephrotoxins. Toxins, 7(4), 1005–1017. 10.3390/toxins7041005 25811304 PMC4417951

[fsn33529-bib-0133] Mantle, P. G. (2002). Risk assessment and the importance of ochratoxins. International Biodeterioration and Biodegradation, 50(3–4), 143–146. 10.1016/S0964-8305(02)00079-3

[fsn33529-bib-0134] Marangoni, C. , & De Moura, N. F. (2011). Antioxidant activity of essential oil from *Coriandrum Sativum* L. in Italian salami. Ciência e Tecnologia de Alimentos, 31(1), 124–128. 10.1590/s0101-20612011000100017

[fsn33529-bib-0135] Marefati, N. , Ghorani, V. , Shakeri, F. , Boskabady, M. , Kianian, F. , Rezaee, R. , & Boskabady, M. H. (2021). A review of anti‐inflammatory, antioxidant, and immunomodulatory effects of *Allium cepa* and its main constituents. Pharmaceutical Biology, 59(1), 287–302. 10.1080/13880209.2021.1874028 33645419 PMC7919894

[fsn33529-bib-0136] Marques, J. d. L. , Volcão, L. M. , Funck, G. D. , Kroning, I. S. , da Silva, W. P. , Fiorentini, Â. M. , & Ribeiro, G. A. (2015). Antimicrobial activity of essential oils of *Origanum vulgare* L. and *Origanum majorana* L. against *Staphylococcus aureus* isolated from poultry meat. Industrial Crops and Products, 77, 444–450. 10.1016/j.indcrop.2015.09.013

[fsn33529-bib-0137] Martins, N. , Petropoulos, S. , & Ferreira, I. C. F. R. (2016). Chemical composition and bioactive compounds of garlic (*Allium sativum* L.) as affected by pre‐ and post‐harvest conditions: A review. Food Chemistry, 211, 41–50. 10.1016/j.foodchem.2016.05.029 27283605

[fsn33529-bib-0138] Mathew, S. , & Abraham, T. E. (2006). Studies on the antioxidant activities of cinnamon (*Cinnamomum verum*) bark extracts, through various *in vitro* models. Food Chemistry, 94(4), 520–528. 10.1016/j.foodchem.2004.11.043

[fsn33529-bib-0139] Mathlouthi, M. (2003). Encyclopedia of food sciences and nutrition (pp. 1918–1922). Academic Press. 10.1016/b0-12-227055-x/00369-2

[fsn33529-bib-0140] Medina, M. Á. T. , Merinas‐Amo, T. , Fernández‐Bedmar, Z. , Font, R. , del Río‐Celestino, M. , Pérez‐Aparicio, J. , Moreno‐Ortega, A. , Alonso‐Moraga, Á. , & Moreno‐Rojas, R. (2019). Physicochemical characterization and biological activities of black and white garlic: *in vivo* and *in vitro* assays. Food, 8(6), 220. 10.3390/foods8060220 PMC6617303 31234387

[fsn33529-bib-0141] Messier, C. , & Grenier, D. (2011). Effect of licorice compounds licochalcone a, glabridin and glycyrrhizic acid on growth and virulence properties of *Candida albicans* . Mycoses, 54(6), 801–806. 10.1111/j.1439-0507.2011.02028.x 21615543

[fsn33529-bib-0142] Miceli, A. , Aleo, A. , Corona, O. , Sardina, M. T. , Mammina, C. , & Settanni, L. (2014). Antibacterial activity of *Borago officinalis* and *Brassica juncea* aqueous extracts evaluated *in vitro* and *in situ* using different food model systems. Food Control, 40(1), 157–164. 10.1016/j.foodcont.2013.12.006

[fsn33529-bib-0143] Mimica‐Dukic, N. , & Bozin, B. (2008). *Mentha* L. species (Lamiaceae) as promising sources of bioactive secondary metabolites. Current Pharmaceutical Design, 14(29), 3141–3150. 10.2174/138161208786404245 19075696

[fsn33529-bib-0144] Mimica‐Dukic, N. , Bozin, B. , Sokovic, M. , Mihajlovic, B. , & Matavulj, M. (2003). Antimicrobial and antioxidant activities of three *Mentha* species essential oils. Planta Medica, 69(5), 413–419. 10.1055/s-2003-39704 12802721

[fsn33529-bib-0145] Miraj, S. , & Alesaeidi, S. (2016). A systematic review study of therapeutic effects of *Matricaria recuitta chamomile* (chamomile). Electronic Physician, 8(9), 3024–3031. 10.19082/3024 27790360 PMC5074766

[fsn33529-bib-0146] Modarresi‐Chahardehi, A. , Ibrahim, D. , Sulaiman, S. F. , & Mousavi, L. (2012). Screening antimicrobial activity of various extracts of *Urtica dioica* . Revista de Biologia Tropical, 60(4), 1567–1576. 10.15517/rbt.v60i4.2074 23342511

[fsn33529-bib-0147] Moghrovyan, A. , Sahakyan, N. , Babayan, A. , Chichoyan, N. , Petrosyan, M. , & Trchounian, A. (2019). Essential oil and ethanol extract of oregano (*Origanum vulgare* L.) from armenian flora as a natural source of terpenes, flavonoids and other phytochemicals with antiradical, antioxidant, metal chelating, tyrosinase inhibitory and antibacterial activity. Current Pharmaceutical Design, 25(16), 1809–1816. 10.2174/1381612825666190702095612 31267860

[fsn33529-bib-0148] Mohamad, R. H. , El‐Bastawesy, A. M. , Abdel‐Monem, M. G. , Noor, A. M. , Al‐Mehdar, H. A. R. , Sharawy, S. M. , & El‐Merzabani, M. M. (2011). Antioxidant and anticarcinogenic effects of methanolic extract and volatile oil of fennel seeds (*Foeniculum vulgare*). Journal of Medicinal Food, 14(9), 986–1001. 10.1089/jmf.2008.0255 21812646

[fsn33529-bib-0149] Mohammed, C. B. , Chahid, B. , Fatima, B. , Fatima‐Zohra, S. , Meriem, B. , & Farid, C. (2014). Antioxidant activity, phenolic and flavonoid content in leaves, flowers, stems and seeds of mallow (*Malva sylvestris* L.) from North Western of Algeria. African Journal of Biotechnology, 13(3), 486–491. 10.5897/ajb2013.12833

[fsn33529-bib-0150] Morales, P. , Carvalho, A. M. , Sánchez‐Mata, M. C. , Cámara, M. , Molina, M. , & Ferreira, I. C. F. R. (2012). Tocopherol composition and antioxidant activity of Spanish wild vegetables. Genetic Resources and Crop Evolution, 59(5), 851–863. 10.1007/s10722-011-9726-1

[fsn33529-bib-0151] Mousavi, S. M. , Hashemi, S. A. , Behbudi, G. , Mazraedoost, S. , Omidifar, N. , Gholami, A. , Chiang, W. H. , Babapoor, A. , & Pynadathu Rumjit, N. (2021). A review on health benefits of *Malva sylvestris* L. nutritional compounds for metabolites, antioxidants, and anti‐inflammatory, anticancer, and antimicrobial applications. Evidence‐Based Complementary and Alternative Medicine, 2021, 1–13. 10.1155/2021/5548404 PMC8382527 34434245

[fsn33529-bib-0152] Msagati, T. A. M. (2013). Chemistry of food additives and preservatives (1st ed., pp. 224–243). John Wiley & Sons, Ltd. 10.1002/9781118274132

[fsn33529-bib-0153] Namshir, J. , Shatar, A. , Khandaa, O. , Tserennadmid, R. , Shiretorova, V. G. , & Nguyen, M. C. (2020). Antimicrobial, antioxidant and cytotoxic activity on human breast cancer cells of essential oil from *Pinus sylvestris*. var *mongolica* needle. Mongolian Journal of Chemistry, 21(47), 19–26. 10.5564/mjc.v21i47.1428

[fsn33529-bib-0154] Narangerel, T. , Bonikowski, R. , Jastrz, K. , Kunicka‐styczy, A. , Smigielski, K. , Sójka, M. , Bonikowski, R. , Jastrząbek, K. , Sroczyński, W. , Plucińska, A. , Kunicka‐Styczyńska, A. , Śmigielski, K. , Majak, I. , Bartos, A. , Leszczyńska, J. , Jastrz, K. , Kunicka‐styczy, A. , & Smigielski, K. (2021). Chemical and biological characteristics of *Oxytropis pseudoglandulosa* plant of Mongolian origin. Molecules, 26(24), 7573. 10.3390/molecules26247573 34946654 PMC8705308

[fsn33529-bib-0155] Narangerel, T. , Sójka, M. , Bonikowski, R. , Jastrząbek, K. , Sroczyński, W. , Plucińska, A. , Kunicka‐Styczyńska, A. , Śmigielski, K. , Majak, I. , Bartos, A. , & Leszczyńska, J. (2021). Chemical and biological profile and allergenicity of *Thymus baicalensis* plant of Mongolian origin. Antioxidants, 10, 1905. 10.3390/antiox10121905 34943008 PMC8750244

[fsn33529-bib-0156] Neidhart, M. (2016). DNA methylation and complex human disease (pp. 405–418). Elsevier Ltd. 10.1016/b978-0-12-420194-1.00025-7

[fsn33529-bib-0157] Nimse, S. B. , & Pal, D. (2015). Free radicals, natural antioxidants, and their reaction mechanisms. RSC Advances, 5(35), 27986–28006. 10.1039/c4ra13315c

[fsn33529-bib-0158] Nishimura, H. , Higuchi, O. , Tateshita, K. , Tomobe, K. , Okuma, Y. , & Nomura, Y. (2006). Antioxidative activity and ameliorative effects of memory impairment of sulfur‐containing compounds in *allium* species. BioFactors, 26(2), 135–146. 10.1002/biof.5520260204 16823099

[fsn33529-bib-0159] Nurzynska‐Wierdak, R. , & Zawislak, G. (2014). Herb yield and bioactive compounds of tarragon (*Artemisia dracunculus* L.) as influenced by plant density. Acta Science Polonorum, 13(2), 207–221.

[fsn33529-bib-0160] Nzeako, B. C. , Al‐Kharousi, Z. S. N. , & Al‐Mahrooqui, Z. (2006). Antimicrobial activities of clove and thyme extracts. Sultan Qaboos University Medical Journal, 6(1), 33–39. PMC3074903 21748125

[fsn33529-bib-0161] Obolskiy, D. , Pischel, I. , Feistel, B. , Glotov, N. , & Heinrich, M. (2011). *Artemisia dracunculus* L. (tarragon): A critical review of its traditional use, chemical composition, pharmacology, and safety. Journal of Agricultural and Food Chemistry, 59(21), 11367–11384. 10.1021/jf202277w 21942448

[fsn33529-bib-0162] Odontuya, G. , & Nomin, M. (2018). Review analysis on phytochemical and biological activity studies on plants of *aquilegia* L. Bulletin of the Institute of Chemistry and Chemical Technology, 6, 64–71. 10.5564/bicct.v0i6.1103

[fsn33529-bib-0163] Oguro, Y. , Yamazaki, H. , Takagi, M. , & Takaku, H. (2014). Antifungal activity of plant defensin AFP1 in *Brassica juncea* involves the recognition of the methyl residue in glucosylceramide of target pathogen *Candida albicans* . Current Genetics, 60(2), 89–97. 10.1007/s00294-013-0416-8 24253293

[fsn33529-bib-0164] Olennikov, D. N. , & Chirikova, N. K. (2013). Phenolic compounds and cinnamamide from *Scutellaria scordiifolia* . Chemistry of Natural Compounds, 49(1), 124–126. 10.1007/s10600-013-0528-x

[fsn33529-bib-0165] Oniga, I. , Pus, C. , Silaghi‐Dumitrescu, R. , Olah, N. K. , Sevastre, B. , Marica, R. , Marcus, I. , Sevastre‐Berghian, A. C. , Benedec, D. , Pop, C. E. , & Hanganu, D. (2018). *Origanum vulgare* ssp. *vulgare*: Chemical composition and biological studies. Molecules, 23(8), 2077. 10.3390/molecules23082077 30126246 PMC6222339

[fsn33529-bib-0166] Paaver, U. , Orav, A. , Arak, E. , Mäeorg, U. , & Raal, A. (2008). Phytochemical analysis of the essential oil of *Thymus serpyllum* L. growing wild in Estonia. Natural Product Research, 22(2), 108–115. 10.1080/14786410601035118 18075894

[fsn33529-bib-0167] Pandey, A. K. , Rai, M. K. , & Acharya, D. (2003). Chemical composition and antimycotic activity of the essential oils of corn mint (*Mentha arvensis*) and lemon grass (*Cymbopogon flexuosus*) against human pathogenic fungi. Pharmaceutical Biology, 41(6), 421–425. 10.1076/phbi.41.6.421.17825

[fsn33529-bib-0168] Parham, S. , Kharazi, A. Z. , Bakhsheshi‐Rad, H. R. , Nur, H. , Ismail, A. F. , Sharif, S. , Ramakrishna, S. , & Berto, F. (2020). Antioxidant, antimicrobial and antiviral properties of herbal materials. Antioxidants, 9(12), 1–36. 10.3390/antiox9121309 PMC7767362 33371338

[fsn33529-bib-0169] Park, S. Y. , Jang, H. L. , Lee, J. H. , Choi, Y. , Kim, H. , Hwang, J. , Seo, D. , Kim, S. , & Nam, J. S. (2017). Changes in the phenolic compounds and antioxidant activities of mustard leaf (*Brassica juncea*) kimchi extracts during different fermentation periods. Food Science and Biotechnology, 26(1), 105–112. 10.1007/s10068-017-0014-5 30263516 PMC6049491

[fsn33529-bib-0170] Parra, C. , Muñoz, P. , Bustos, L. , Parra, F. , Simirgiotis, M. J. , & Escobar, H. (2021). UHPLC‐DAD characterization of *Origanum vulgare* L. from Atacama desert Andean region and antioxidant, antibacterial and enzyme inhibition activities. Molecules, 26(7), 2100. 10.3390/molecules26072100 33917599 PMC8038783

[fsn33529-bib-0171] Pârvu, M. , Moţ, C. A. , Pârvu, A. E. , Mircea, C. , Stoeber, L. , Roşca‐Casian, O. , & Ţigu, A. B. (2019). *Allium sativum* extract chemical composition, antioxidant activity and antifungal effect against *Meyerozyma guilliermondii* and *Rhodotorula mucilaginosa* causing onychomycosis. Molecules, 24(21), 1–16. 10.3390/molecules24213958 PMC6865177 31683743

[fsn33529-bib-0172] Pârvu, M. , Pârvu, A. E. , Vlase, L. , Rosca‐Casian, O. , Pârvu, O. , & Puşcaş, M. (2011). Allicin and alliin content and antifungal activity of *Allium senescens* L. ssp. *montanum* (F. W. Schmidt) Holub ethanol extract. Journal of Medicinal Plant Research, 5(29), 6544–6549. 10.5897/JMPR11.818

[fsn33529-bib-0173] Pastorino, G. , Cornara, L. , Soares, S. , Rodrigues, F. , & Oliveira, M. B. P. P. (2018). Liquorice (*Glycyrrhiza glabra*): A phytochemical and pharmacological review. Phytotherapy Research, 32(12), 2323–2339. 10.1002/ptr.6178 30117204 PMC7167772

[fsn33529-bib-0174] Pezzani, R. , Vitalini, S. , & Iriti, M. (2017). Bioactivities of *Origanum vulgare* L.: An update. Phytochemistry Reviews, 16(6), 1253–1268. 10.1007/s11101-017-9535-z

[fsn33529-bib-0175] Pitaro, S. , Fiorani, L. , & Jorge, N. (2013). Antioxidant activity of basil and oregano extracts added to soybean oil for accelerated storage test. Journal of Food Biochemistry, 37(4), 485–490. 10.1111/j.1745-4514.2012.00653.x

[fsn33529-bib-0176] Pitschmann, A. , Purevsuren, S. , Obmann, A. , Natsagdorj, D. , Gunbilig, D. , Narantuya, S. , Kletter, C. , & Glasl, S. (2013). Traditional Mongolian medicine: History and status quo. Phytochemistry Reviews, 12(4), 943–959. 10.1007/s11101-013-9321-5

[fsn33529-bib-0177] Pitt, J. I. , & Hocking, A. D. (2013). Fungi and food spoilage (1st ed.). Springer. 10.1007/978-0-387-92207-2

[fsn33529-bib-0178] Potente, G. , Bonvicini, F. , Gentilomi, G. A. , & Antognoni, F. (2020). Anti‐*Candida* activity of essential oils from Lamiaceae plants from the Mediterranean area and the Middle East. Antibiotics, 9(7), 1–26. 10.3390/antibiotics9070395 PMC7400371 32660009

[fsn33529-bib-0179] Prados, J. , Melguizo, C. , Aranega, A. , Boulaiz, H. , & Marchal, J. (2005). Cancer gene therapy: Strategies and clinical trials. Cellular and Molecular Biology, 51(1), 23–36 https://europepmc.org/article/med/16171562 16171562

[fsn33529-bib-0180] Prakash, D. , Singh, B. N. , & Upadhyay, G. (2007). Antioxidant and free radical scavenging activities of phenols from onion (*Allium cepa*). Food Chemistry, 102(4), 1389–1393. 10.1016/j.foodchem.2006.06.063

[fsn33529-bib-0181] Prakash, V. , Kumari, A. , Kaur, H. , Kumar, M. , Gupta, S. , & Bala, R. (2021). Chemical composition and antimicrobial activity of essential oil from aerial parts of *Origanum vulgare* L. from North‐Western Himalayas. Journal of Essential Oil‐Bearing Plants, 24(2), 177–185. 10.1080/0972060X.2021.1919928

[fsn33529-bib-0182] Purcell, S. C. , Pande, P. , Lin, Y. , Rivera, E. J. , Latisha, P. U. , Smallwood, L. M. , Kerstiens, G. A. , Armstrong, L. B. , Robak, M. T. , Baranger, A. M. , & Douskey, M. C. (2016). Extraction and antibacterial properties of thyme leaf extracts: Authentic practice of green chemistry. Journal of Chemical Education, 93(8), 1422–1427. 10.1021/acs.jchemed.5b00891

[fsn33529-bib-0183] Qin, L. , Yu, J. , Zhu, J. , Kong, B. , & Chen, Q. (2021). Ultrasonic‐assisted extraction of polyphenol from the seeds of *Allium senescens* L. and its antioxidative role in Harbin dry sausage. Meat Science, 172(August 2020), 108351. 10.1016/j.meatsci.2020.108351 33120179

[fsn33529-bib-0184] Rahimi, V. B. , Ajam, F. , Rakhshandeh, H. , & Askari, V. R. (2019). A pharmacological review on *Portulaca oleracea* L.: Focusing on antiinflammatory, antioxidant, immunomodulatory and antitumor activities. Journal of Pharmacopuncture, 22(1), 7–15. 10.3831/KPI.2019.22.001 30988996 PMC6461301

[fsn33529-bib-0185] Rasooli, I. , & Allameh, A. (2016). Essential oils in food preservation, flavor and safety (pp. 287–293). Elsevier Inc. 10.1016/B978-0-12-416641-7.00032-8

[fsn33529-bib-0186] Razavi, S. M. , Zarrini, G. , Molavi, G. , & Ghasemi, G. (2011). Bioactivity of *Malva sylvestris* L., a medicinal plant from Iran. Iranian Journal of Basic Medical Sciences, 14(6), 574–579. 10.22038/ijbms.2011.5058 23493458 PMC3586856

[fsn33529-bib-0187] Roby, M. H. H. , Sarhan, M. A. , Selim, K. A. H. , & Khalel, K. I. (2013). Antioxidant and antimicrobial activities of essential oil and extracts of fennel (*Foeniculum vulgare* L.) and chamomile (*Matricaria chamomilla* L.). Industrial Crops and Products, 44, 437–445. 10.1016/j.indcrop.2012.10.012

[fsn33529-bib-0188] Salehi, B. , Stojanović‐Radić, Z. , Matejić, J. , Sharopov, F. , Antolak, H. , Kręgiel, D. , Sen, S. , Sharifi‐Rad, M. , Acharya, K. , Sharifi‐Rad, R. , Martorell, M. , Sureda, A. , Martins, N. , & Sharifi‐Rad, J. (2018). Plants of genus *Mentha*: From farm to food factory. Plants, 7(3), 70. 10.3390/plants7030070 30181483 PMC6161068

[fsn33529-bib-0189] Salehzadeh, A. , Asadpour, L. , Naeemi, A. S. , & Houshmand, E. (2014). Antimicrobial activity of methanolic extracts of *Sambucus ebulus* and *Urtica dioica* against clinical isolates of methicillin resistant *Staphylococcus aureus* . African Journal of Traditional, Complementary and Alternative Medicines, 11(5), 38–40. 10.4314/ajtcam.v11i5.6 PMC4202515 25395702

[fsn33529-bib-0190] Samojlik, I. , Lakić, N. , Mimica‐Dukić, N. , Daković‐Švajcer, K. , & Božin, B. (2010). Antioxidant and hepatoprotective potential of essential oils of coriander (*Coriandrum sativum* L.) and caraway (*Carum carvi* L.) (Apiaceae). Journal of Agricultural and Food Chemistry, 58(15), 8848–8853. 10.1021/jf101645n 20608729

[fsn33529-bib-0191] Sánchez, M. , González‐Burgos, E. , & Gómez‐Serranillos, M. P. (2022). The pharmacology and clinical efficacy of *Matricaria recutita* L.: A systematic review of *in vitro*, *in vivo* studies and clinical trials. Food Reviews International, 38(8), 1668–1702. 10.1080/87559129.2020.1834577

[fsn33529-bib-0192] Sandulachi, E. , Macari, A. , Ghendov‐Mosanu, A. , Cojocari, D. , & Sturza, R. (2021). Antioxidant and antimicrobial activity of basil, thyme and tarragon used in meat products. Advances in Microbiology, 11(11), 591–606. 10.4236/aim.2021.1111043

[fsn33529-bib-0193] Sanzani, S. M. , Reverberi, M. , & Geisen, R. (2016). Mycotoxins in harvested fruits and vegetables: Insights in producing fungi, biological role, conducive conditions, and tools to manage postharvest contamination. Postharvest Biology and Technology, 122, 95–105. 10.1016/j.postharvbio.2016.07.003

[fsn33529-bib-0194] Saruul, E. , Murata, T. , Selenge, E. , Sasaki, K. , Yoshizaki, F. , & Batkhuu, J. (2015). An antibacterial ortho‐quinone diterpenoid and its derivatives from *Caryopteris mongolica* . Bioorganic and Medicinal Chemistry Letters, 25(12), 2555–2558. 10.1016/j.bmcl.2015.04.048 25958242

[fsn33529-bib-0195] Sayed‐Ahmad, B. , Straumite, E. , Šabovics, M. , Kruma, Z. , Merah, O. , Saad, Z. , Hijazi, A. , & Talou, T. (2017). Effect of addition of fennel (*Foeniculum vulgare* L.) on the quality of protein bread. Proceedings of the Latvian Academy of Sciences, Section B: Natural, Exact, and Applied Sciences, 71(6), 509–514. 10.1515/prolas-2017-0088

[fsn33529-bib-0196] Scalas, D. , Mandras, N. , Roana, J. , Tardugno, R. , Cuffini, A. M. , Ghisetti, V. , Benvenuti, S. , & Tullio, V. (2018). Use of *Pinus sylvestris* L. (*Pinaceae*), *Origanum vulgare* L. (*Lamiaceae*), and *Thymus vulgaris* L. (*Lamiaceae*) essential oils and their main components to enhance itraconazole activity against azole susceptible/not‐susceptible *Cryptococcus neoformans* strains. BMC Complementary and Alternative Medicine, 18(1), 1–13. 10.1186/s12906-018-2219-4 29724221 PMC5934896

[fsn33529-bib-0197] Sefidkon, F. , Dabiri, M. , & Mirmostafa, S. A. (2004). The composition of *Thymus serpyllum* L. oil. Journal of Essential Oil Research, 16(3), 184–185. 10.1080/10412905.2004.9698691

[fsn33529-bib-0198] Seidler‐Łozykowska, K. , Kedzia, B. , Karpińska, E. , & Bocianowski, J. (2013). A atividade microbiológica do óleo essencial de alcaravia (*Carum carvi* L.) de várias origens. Acta Scientiarum–Agronomy, 35(4), 495–500. 10.4025/actasciagron.v35i4.16900

[fsn33529-bib-0199] Shahat, A. A. , Ibrahim, A. Y. , Hendawy, S. F. , Omer, E. A. , Hammouda, F. M. , Abdel‐Rahman, F. H. , & Saleh, M. A. (2011). Chemical composition, antimicrobial and antioxidant activities of essential oils from organically cultivated fennel cultivars. Molecules, 16(2), 1366–1377. 10.3390/molecules16021366 21285921 PMC6259638

[fsn33529-bib-0200] Shams‐Ghahfarokhi, M. , Shokoohamiri, M. R. , Amirrajab, N. , Moghadasi, B. , Ghajari, A. , Zeini, F. , Sadeghi, G. , & Razzaghi‐Abyaneh, M. (2006). *In vitro* antifungal activities of *Allium cepa*, *Allium sativum* and ketoconazole against some pathogenic yeasts and dermatophytes. Fitoterapia, 77(4), 321–323. 10.1016/j.fitote.2006.03.014 16690223

[fsn33529-bib-0201] Shan, B. , Cai, Y. Z. , Sun, M. , & Corke, H. (2005). Antioxidant capacity of 26 spice extracts and characterization of their phenolic constituents. Journal of Agricultural and Food Chemistry, 53(20), 7749–7759. 10.1021/jf051513y 16190627

[fsn33529-bib-0202] Shang, X. , He, X. , He, X. , Li, M. , Zhang, R. , Fan, P. , Zhang, Q. , & Jia, Z. (2010). The genus *Scutellaria* an ethnopharmacological and phytochemical review. Journal of Ethnopharmacology, 128(2), 279–313. 10.1016/j.jep.2010.01.006 20064593

[fsn33529-bib-0203] Sharifi‐Rad, J. , Melgar‐Lalanne, G. , Hernández‐Álvarez, A. J. , Taheri, Y. , Shaheen, S. , Kregiel, D. , Antolak, H. , Pawlikowska, E. , Brdar‐Jokanović, M. , Rajkovic, J. , Hosseinabadi, T. , Ljevnaić‐Mašić, B. , Baghalpour, N. , Mohajeri, M. , Fokou, P. V. T. , & Martins, N. (2020). *Malva* species: Insights on its chemical composition towards pharmacological applications. Phytotherapy Research, 34(3), 546–567. 10.1002/ptr.6550 31713320

[fsn33529-bib-0204] Sharifi‐Rad, M. , Nazaruk, J. , Polito, L. , Morais‐Braga, M. F. B. , Rocha, J. E. , Coutinho, H. D. M. , Salehi, B. , Tabanelli, G. , Montanari, C. , del Mar Contreras, M. , Yousaf, Z. , Setzer, W. N. , Verma, D. R. , Martorell, M. , Sureda, A. , & Sharifi‐Rad, J. (2018). *Matricaria* genus as a source of antimicrobial agents: From farm to pharmacy and food applications. Microbiological Research, 215(June), 76–88. 10.1016/j.micres.2018.06.010 30172312

[fsn33529-bib-0205] Sharma, K. , Assefa, A. D. , Kim, S. , Ko, E. Y. , Lee, E. T. , & Park, S. W. (2014). Evaluation of total phenolics, flavonoids and antioxidant activity of 18 Korean onion cultivars: A comparative study. Journal of the Science of Food and Agriculture, 94(8), 1521–1529. 10.1002/jsfa.6450 24136245

[fsn33529-bib-0206] Sicari, V. , Loizzo, M. R. , Tundis, R. , Mincione, A. , & Pellicanò, T. M. (2018). *Portulaca oleracea* L. (purslane) extracts display antioxidant and hypoglycaemic effects. Journal of Applied Botany and Food Quality, 91, 39–46. 10.5073/JABFQ.2018.091.006

[fsn33529-bib-0207] Simmler, C. , Pauli, G. F. , & Chen, S. N. (2013). Phytochemistry and biological properties of glabridin. Fitoterapia, 90, 160–184. 10.1016/j.fitote.2013.07.003 23850540 PMC3795865

[fsn33529-bib-0208] Singh, O. , Khanam, Z. , Misra, N. , & Srivastava, M. K. (2011). Chamomile (*Matricaria chamomilla* L.): An overview. Pharmacognosy Reviews, 5(9), 82–95. 10.4103/0973-7847.79103 22096322 PMC3210003

[fsn33529-bib-0209] Singh, P. , & Pandey, A. K. (2018). Prospective of essential oils of the genus *Mentha* as biopesticides: A review. Frontiers in Plant Science, 9(September), 1–14. 10.3389/fpls.2018.01295 30250476 PMC6139362

[fsn33529-bib-0210] Singh, V. , Pal, A. , & Darokar, M. P. (2015). A polyphenolic flavonoid glabridin: Oxidative stress response in multidrug‐resistant *Staphylococcus aureus* . Free Radical Biology and Medicine, 87, 48–57. 10.1016/j.freeradbiomed.2015.06.016 26117328

[fsn33529-bib-0211] Siracusa, L. , Saija, A. , Cristani, M. , Cimino, F. , D'Arrigo, M. , Trombetta, D. , Rao, F. , & Ruberto, G. (2011). Phytocomplexes from liquorice (*Glycyrrhiza glabra* L.) leaves–chemical characterization and evaluation of their antioxidant, anti‐genotoxic and anti‐inflammatory activity. Fitoterapia, 82(4), 546–556. 10.1016/j.fitote.2011.01.009 21281704

[fsn33529-bib-0212] Siriamornpun, S. , & Suttajit, M. (2010). Microchemical components and antioxidant activity of different morphological parts of Thai wild purslane (*Portulaca oleracea*). Weed Science, 58(3), 182–188. 10.1614/ws-d-09-00073.1

[fsn33529-bib-0213] Sivapalan, S. R. (2016). Biological and pharmacological studies of *Tribulus terrestris* Linn‐ a review. International Journal of Multidisciplinary Research and Development, 3(1), 257–265.

[fsn33529-bib-0214] Soltani, S. , Shakeri, A. , Iranshahi, M. , & Boozari, M. (2021). A review of the phytochemistry and antimicrobial properties of *Origanum vulgare* L. and subspecies. Iranian Journal of Pharmaceutical Research, 20(2), 268–285. 10.22037/ijpr.2020.113874.14539 34567161 PMC8457725

[fsn33529-bib-0215] Souri, E. , Amin, G. , Farsam, H. , & Andaji, S. (2004). The antioxidant activity of some commonly used vegetables in Iranian diet. Fitoterapia, 75(6), 585–588. 10.1016/j.fitote.2004.04.007 15351114

[fsn33529-bib-0216] Sriti, J. , Wannes, W. A. , Talou, T. , Vilarem, G. , & Marzouk, B. (2011). Chemical composition and antioxidant activities of Tunisian and Canadian coriander (*Coriandrum sativum* L.) fruit. Journal of Essential Oil Research, 23(4), 7–15. 10.1080/10412905.2011.9700462

[fsn33529-bib-0217] Stanojevic, L. P. , Marjanovic‐Balaban, Z. R. , Kalaba, V. D. , Stanojevic, J. S. , & Cvetkovic, D. J. (2016). Chemical composition, antioxidant and antimicrobial activity of chamomile flowers essential oil (*Matricaria chamomilla* L.). Journal of Essential Oil‐Bearing Plants, 19(8), 2017–2028. 10.1080/0972060X.2016.1224689

[fsn33529-bib-0218] Sturm, C. , & Wagner, A. E. (2017). *Brassica*‐derived plant bioactives as modulators of chemopreventive and inflammatory signaling pathways. International Journal of Molecular Sciences, 18(9), 1890. 10.3390/ijms18091890 28862664 PMC5618539

[fsn33529-bib-0219] Sun, B. , Tian, Y. X. , Jiang, M. , Yuan, Q. , Chen, Q. , Zhang, Y. , Luo, Y. , Zhang, F. , & Tang, H. R. (2018). Variation in the main health‐promoting compounds and antioxidant activity of whole and individual edible parts of baby mustard (*Brassica juncea* var. *gemmifera*). RSC Advances, 8(59), 33845–33854. 10.1039/C8RA05504A 35548826 PMC9086739

[fsn33529-bib-0220] Szychowski, K. A. , Rybczyńska‐Tkaczyk, K. , Gaweł‐Bȩben, K. , Ašwieca, M. , Kara, M. , Jakubczyk, A. , Matysiak, M. , Binduga, U. E. , & Gmiński, J. (2018). Characterization of active compounds of different garlic (*Allium sativum* L.) cultivars. Polish Journal of Food and Nutrition Sciences, 68(1), 73–81. 10.1515/pjfns-2017-0005

[fsn33529-bib-0221] Tafrihi, M. , Imran, M. , Tufail, T. , Gondal, T. A. , Caruso, G. , Sharma, S. , Sharma, R. , Atanassova, M. , Atanassov, L. , Fokou, P. V. T. , & Pezzani, R. (2021). The wonderful activities of the genus *Mentha*: Not only antioxidant properties. Molecules, 26(4), 1–22. 10.3390/molecules26041118 PMC7923432 33672486

[fsn33529-bib-0222] Teixeira, B. , Marques, A. , Ramos, C. , Serrano, C. , Matos, O. , Neng, N. R. , Nogueira, J. M. F. , Saraiva, J. A. , & Nunes, M. L. (2013). Chemical composition and bioactivity of different oregano (*Origanum vulgare*) extracts and essential oil. Journal of the Science of Food and Agriculture, 93(11), 2707–2714. 10.1002/jsfa.6089 23553824

[fsn33529-bib-0223] Thiyam, U. , Stöckmann, H. , & Schwarz, K. (2006). Antioxidant activity of rapeseed phenolics and their interactions with tocopherols during lipid oxidation. Journal of the American Oil Chemists' Society, 83(6), 523–528. 10.1007/s11746-006-1235-6

[fsn33529-bib-0224] Thiyam, U. , Stöckmann, H. , Zum Felde, T. , & Schwarz, K. (2006). Antioxidative effect of the main sinapic acid derivatives from rapeseed and mustard oil by‐products. European Journal of Lipid Science and Technology, 108(3), 239–248. 10.1002/ejlt.200500292

[fsn33529-bib-0225] Tian, Y. , & Deng, F. (2020). Phytochemistry and biological activity of mustard (*Brassica juncea*): A review. CyTA–Journal of Food, 18(1), 704–718. 10.1080/19476337.2020.1833988

[fsn33529-bib-0226] Tomović, V. , Šojić, B. , Savanović, J. , Kocić‐Tanackov, S. , Pavlić, B. , Jokanović, M. , Đorđević, V. , Parunović, N. , Martinović, A. , & Vujadinović, D. (2022). Caraway (*Carum carvi* L.) essential oil improves quality of dry‐fermented sausages produced with different levels of sodium nitrite. Journal of Food Processing and Preservation, 46(10), e15786. 10.1111/jfpp.15786

[fsn33529-bib-0227] Tsivelika, N. , Irakli, M. , Mavromatis, A. , Chatzopoulou, P. , & Karioti, A. (2021). Phenolic profile by HPLC‐PDA‐MS of Greek chamomile populations and commercial varieties and their antioxidant activity. Food, 10(10), 2345. 10.3390/foods10102345 PMC8535277 34681394

[fsn33529-bib-0228] Varma, J. , & Dubey, N. K. (2001). Efficacy of essential oils of *Caesulia axillaris* and *Mentha arvensis* against some storage pests causing biodeterioration of food commodities. International Journal of Food Microbiology, 68(3), 207–210. 10.1016/S0168-1605(01)00506-2 11529443

[fsn33529-bib-0229] Wang, W. , Li, J. , Zhang, H. , Wang, X. , Fan, J. , & Zhang, X. (2019). Phenolic compounds and bioactivity evaluation of aqueous and methanol extracts of *Allium mongolicum* regel. Food Science and Nutrition, 7(2), 779–787. 10.1002/fsn3.926 30847157 PMC6392871

[fsn33529-bib-0230] Wang, Z. F. , Wang, B. B. , Zhao, Y. , Wang, F. X. , Sun, Y. , Guo, R. J. , Song, X. B. , Xin, H. L. , & Sun, X. G. (2016). Furostanol and spirostanol saponins from *Tribulus terrestris* . Molecules, 21(4), 3–16. 10.3390/molecules21040429 PMC6273128 27043512

[fsn33529-bib-0231] Węglarz, Z. , Kosakowska, O. , Przybył, J. L. , Pióro‐Jabrucka, E. , & Bączek, K. (2020). The quality of Greek oregano (*O. vulgare* L. subsp. *hirtum* (link) Ietswaart) and common oregano (*O. vulgare L*. subsp. *vulgare*) cultivated in the temperate climate of Central Europe. Food, 9(11), 1671. 10.3390/foods9111671 PMC7697828 33203184

[fsn33529-bib-0232] WHO . (2008). Safety evaluation of certain food additives and contaminants . https://apps.who.int/food‐additives‐contaminants‐jecfa‐database/chemical.aspx?chemID=1905

[fsn33529-bib-0233] WHO . (2013). Medicinal plants in Mongolia . https://apps.who.int/iris/bitstream/handle/10665/207671/9789290616320_eng.pdf

[fsn33529-bib-0234] Woo, K. W. , Moon, E. , Park, S. Y. , Kim, S. Y. , & Lee, K. R. (2012). Flavonoid glycosides from the leaves of *Allium victorialis* var. *platyphyllum* and their anti‐neuroinflammatory effects. Bioorganic and Medicinal Chemistry Letters, 22(24), 7465–7470. 10.1016/j.bmcl.2012.10.043 23149227

[fsn33529-bib-0235] Xu, Y. J. (2017). Foodomics: A novel approach for food microbiology. TrAC–Trends in Analytical Chemistry, 96, 14–21. 10.1016/j.trac.2017.05.012

[fsn33529-bib-0236] Yu, J. , Lei, J. , Yu, H. , Cai, X. , & Zou, G. (2004). Chemical composition and antimicrobial activity of the essential oil of *Scutellaria barbata* . Phytochemistry, 65(7), 881–884. 10.1016/j.phytochem.2004.02.005 15081288

[fsn33529-bib-0237] Zare, M. , Mirzakhani, M. K. , & Stejskal, V. (2021). Growth, body proximate composition and selected blood parameters of rainbow trout *Onchorhynchus mykiss* fingerlings fed on a diet supplemented with nettle *Urtica dioica* and tarragon *Artemisia dracunculus* . Aquaculture Research, 52(11), 5691–5702. 10.1111/are.15444

[fsn33529-bib-0238] Zeeshan Amanullah, K. , Zeenat, F. , Ahmad, W. , Firoz, S. , Naeela, A. , & Author, C. (2021). A comprehensive review on a Unani medicinal plant: *Tribulus terrestris* Linn. International Journal of Herbal Medicine, 9(1), 23–28 https://www.florajournal.com/archives/2021/vol9issue1/PartA/8‐5‐15‐149.pdf

[fsn33529-bib-0239] Zeljković, S. Ć. , Šišková, J. , Komzáková, K. , De Diego, N. , Kaffková, K. , & Tarkowski, P. (2021). Phenolic compounds and biological activity of selected *Mentha* species. Plants, 10(3), 1–18. 10.3390/plants10030550 PMC8000339 33804017

[fsn33529-bib-0240] Zhang, X. L. , Guo, Y. S. , Wang, C. H. , Li, G. Q. , Xu, J. J. , Chung, H. Y. , Ye, W. C. , Li, Y. L. , & Wang, G. C. (2014). Phenolic compounds from *Origanum vulgare* and their antioxidant and antiviral activities. Food Chemistry, 152, 300–306. 10.1016/j.foodchem.2013.11.153 24444941

[fsn33529-bib-0241] Zhen‐yu, W. (2005). Impact of anthocyanin from *Malva sylvestris* on plasma lipids and free radical. Journal of Forestry Research, 16(3), 228–232. 10.1007/bf02856821

[fsn33529-bib-0242] Zhou, Y. X. , Xin, H. L. , Rahman, K. , Wang, S. J. , Peng, C. , & Zhang, H. (2015). *Portulaca oleracea* L.: A review of phytochemistry and pharmacological effects. BioMed Research International, 2015, 925631. 10.1155/2015/925631 25692148 PMC4321094

[fsn33529-bib-0243] Zolboo, B. , Tsenguunmaa, L. , Undram, O. , & Batkhuu, J. (2015). Quorum sensing screening of some medicinal plants from Mongolia. Mongolian Journal of Agricultural Sciences, 13(2), 63–65. 10.5564/mjas.v13i2.518

